# Glial Cells in the Early Stages of Neurodegeneration: Pathogenesis and Therapeutic Targets

**DOI:** 10.3390/ijms262411995

**Published:** 2025-12-12

**Authors:** Eugenia Ahremenko, Alexander Andreev, Danila Apushkin, Eduard Korkotian

**Affiliations:** 1 Faculty of Chemistry, Perm State University, 614068 Perm, Russia; ahremencko.ev@yandex.ru (E.A.); mnium@yandex.ru (A.A.); apushkinjob@gmail.com (D.A.); 2 Institute of Physics and Mathematics, Perm State University, 614068 Perm, Russia; 3 Institute of Physiologically Active Compounds, Federal Research Center of Problems of Chemical Physics and Medicinal Chemistry, Russian Academy of Sciences, 142432 Chernogolovka, Russia; 4 Department of Brain Sciences, The Weizmann Institute of Science, Rehovot 7610001, Israel

**Keywords:** neurodegeneration, glial cells, therapeutic targets, phenotypes, mice models, mitochondrial dysfunction, microglia polarization, phagocytosis, biogenesis pathways

## Abstract

Astrocytes and microglia constitute nearly half of all central nervous system cells and are indispensable for its proper function. Both exhibit striking morphological and functional heterogeneity, adopting either neuroprotective (A2, M2) or proinflammatory (A1, M1) phenotypes in response to cytokines, pathogen-associated molecular patterns (PAMPs)/damage-associated molecular patterns (DAMPs), toll-like receptor 4 (TLR4) activation, and NOD-like receptor family pyrin domain containing 3 (NLRP3) inflammasome signaling. Crucially, many of these phenotypic transitions arise during the earliest stages of neurodegeneration, when glial dysfunction precedes overt neuronal loss and may act as a primary driver of disease onset. This review critically examines glial-centered hypotheses of neurodegeneration, with emphasis on their roles in early disease phases: (i) microglial polarization from an M2 neuroprotective state to an M1 proinflammatory state; (ii) NLRP3 inflammasome assembly via P2X purinergic receptor 7 (P2X7R)-mediated K^+^ efflux; (iii) a self-amplifying astrocyte–microglia–neuron inflammatory feedback loop; (iv) impaired microglial phagocytosis and extracellular-vesicle–mediated propagation of β-amyloid (Aβ) and tau; (v) astrocytic scar formation driven by aquaporin-4 (AQP4), matrix metalloproteinase-9 (MMP-9), glial fibrillary acidic protein (GFAP)/vimentin, connexins, and janus kinase/signal transducer and activator of transcription 3 (JAK/STAT3) signaling; (vi) cellular reprogramming of astrocytes and NG2 glia into functional neurons; and (vii) mitochondrial dysfunction in glia, including Dynamin-related protein 1/Mitochondrial fission protein 1 (Drp1/Fis1) fission imbalance and dysregulation of the sirtuin 1/peroxisome proliferator-activated receptor gamma coactivator 1-alpha (Sirt1/PGC-1α) axis. Promising therapeutic strategies target pattern-recognition receptors (TLR4, NLRP3/caspase-1), cytokine modulators (interleukin-4 (IL-4), interleukin-10 (IL-10)), signaling cascades (JAK2–STAT, nuclear factor kappa-light-chain-enhancer of activated B cells (NF-κB), phosphoinositide 3-kinase–protein kinase B (PI3K–AKT), adenosine monophosphate-activated protein kinase (AMPK)), microglial receptors (triggering receptor expressed on myeloid cells 2 (TREM2)/spleen tyrosine kinase (SYK)/ DNAX-activating protein 10 (DAP10), siglec-3 (CD33), chemokine C-X3-C motif ligand 1/ CX3C motif chemokine receptor 1 (CX3CL1/CX3CR1), Cluster of Differentiation 200/ Cluster of Differentiation 200 receptor 1 (CD200/CD200R), P2X7R), and mitochondrial biogenesis pathways, with a focus on normalizing glial phenotypes rather than simply suppressing pathology. Interventions that restore neuroglial homeostasis at the earliest stages of disease may hold the greatest potential to delay or prevent progression. Given the complexity of glial phenotypes and molecular isoform diversity, a comprehensive, multitargeted approach is essential for mitigating Alzheimer’s disease and related neurodegenerative disorders. This review not only synthesizes pathogenesis but also highlights therapeutic opportunities, offering what we believe to be the first concise overview of the principal hypotheses implicating glial cells in neurodegeneration. Rather than focusing on isolated mechanisms, our goal is a holistic perspective—integrating diverse glial processes to enable comparison across interconnected pathological conditions.

## 1. Introduction

In addition to neurons, glial cells are major components of the central nervous system (CNS), with astrocytes, microglia, and oligodendrocytes together comprising ~50% of all cells [1,2,3]. In the peripheral nervous system (PNS), Schwann and satellite cells predominate. While neurons mediate signal transmission, nervous-system maintenance depends on neuroglia, which are increasingly implicated in neurodegenerative diseases such as Alzheimer’s [3,4,5,6,7,8]. Glial functions include ion homeostasis, structural support, and regulation of the extracellular environment. Astrocytes, the largest glial population (~40%), modulate synaptic activity, support axonal growth, contribute to the blood–brain barrier (BBB), and form glial scars [8,9,10,11]. Microglia (~30%) act as resident immune cells, protecting against infection, clearing debris, and supporting neurogenesis and synaptic plasticity [3,5,12]. Both astrocytes and microglia secrete diverse signaling molecules—neurotransmitters, neuromodulators, hormones, growth factors, and inflammatory mediators—via vesicular transport [3,13,14,15,16].

### 1.1. Subtypes of Astrocytes

Astrocytes, derived from ectodermal neuroepithelial tissue with radial glia as precursors, can be subdivided by morphology and function [17,18,19].

- Protoplasmic astrocytes: most abundant, highly branched, located in cortical layers II–VI of gray matter; form microdomains around vessels and synapses, coupling synaptic activity with blood flow [17,19].
- Fibrous astrocytes: larger, less branched, found in white matter near axons and tracts; regulate ionic and metabolic homeostasis along conduction pathways [17,19].
- Interlaminar astrocytes: primate-specific, compact bodies in cortical layer I with long tangential processes into layers III–IV [17,18,19].
- Varicose projection astrocytes: primate-specific, in cortical layers V–VI; few long peduncular processes with varicosities, possibly linked to higher cognition [8,20].

Additional specialized populations include velate astrocytes (envelop neuronal somata in olfactory bulb and cerebellum) and juxtavascular astrocytes (injury-responsive protoplasmic subtype) [17,19]. Radial astrocytes comprise Bergmann glia (cerebellum), Müller glia (retina), radial astrocytes of the supraoptic nucleus, radial glial stem cells, and tanycytes of hypothalamic/pituitary periventricular regions [21]. In the spinal cord and periphery, astrocytes are classified as Type I (dorsal/ventral spinal regions, optic nerve periphery) and Type II (ventral horns, Nodes of Ranvier) [22].

Astrocytes adapt to stressors such as oxidative imbalance or hypothermia by secreting survival factors [3,15,23,24]. Under trauma or disease, they remodel into reactive phenotypes, often categorized as A1 (neurotoxic/proinflammatory) or A2 (neuroprotective/anti-inflammatory) [25,26]. However, this binary scheme may oversimplify astrocytic diversity, as accurate characterization requires multifaceted molecular and functional assessment, and distinction between pathological remodeling and intrinsic physiological plasticity [25,27].

### 1.2. Subtypes of Microglia

Unlike astrocytes of ectodermal origin, microglia derive from mesodermal erythromyeloid progenitors in the embryonic yolk sac, which migrate into the CNS and mature into resident innate immune cells [28,29]. Microglia display notable plasticity, adopting distinct phenotypes depending on location and environmental cues [30,31,32,33,34]:

- Ramified (resting) microglia: small soma with thin, motile processes that continuously survey neuronal activity and the local milieu [30,32,33,34].
- Hyper-ramified microglia: intermediate between resting and reactive states, with elongated, highly branched processes [32,33,34].
- Activated (reactive) microglia: enlarged soma with shortened, thickened processes; subdivided into M1 (neurotoxic/proinflammatory) and M2 (neuroprotective/anti-inflammatory) forms [30,31,33,34].
- Amoeboid microglia: fully activated, process-free cells resembling macrophages [30,32,33].

Microglia transition through these forms during activation. Resting cells monitor the environment, while early activation produces hyper-ramified morphologies linked to mild responses in early disease. Progression to reactive states triggers cytokine release, and fully activated amoeboid cells engage in phagocytosis. Prolonged phagocytic activity yields enlarged, lipid-laden gitter (reticuloendothelial) cells [31,32,35].

### 1.3. Mechanisms of Astrocyte and Microglia Activation

Several molecular factors activate astrocytes and microglia. Specific cytokines, such as interleukin-4 (IL-4), interleukin-10 (IL-10), and transforming growth factor-β (TGF-β), can induce the A2 astrocyte phenotype or the M2 microglial phenotype, leading to the release of neuroprotective molecules [12,36,37,38]. Table 1 summarizes the brain-related proinflammatory cytokines.

In contrast, proinflammatory molecules—such as pathogen-associated molecular patterns (PAMPs), damage-associated molecular patterns (DAMPs), and certain interleukins—drive astrocytes and microglia toward the A1 and M1 phenotypes, respectively [12,36,39,40]. These reactive phenotypes coordinate defense by recruiting additional microglia and macrophages [41] and by phagocytosing pathological agents. A1 and M1 cells also secrete mediators such as tumor necrosis factor α (TNF-α), interleukins including interleukin-1 beta (IL-1β) and interleukin-6 (IL-16), CC chemokines, and nitric oxide [3,5,7,12,15,42]. The composition of their released extracellular vesicles varies under different conditions [43,44].

Numerous hypotheses have been proposed regarding astrocyte and microglia activation in brain aging and the development of neurodegenerative diseases (NDDs). Reactive astrocytes and microglia are heavily implicated in pathological aging and Alzheimer’s disease, particularly through diverse inflammatory processes [39,45,46,47,48,49]. For example, disrupted iron and copper homeostasis in astrocytes and microglia promotes Fe^2+^/Cu^+^-catalyzed Fenton reactions, generating reactive oxygen species that drive lipid peroxidation and regulated cell death pathways such as ferroptosis and cuproptosis (for details see Section 9). Neuromelanin, which accumulates with age in dopaminergic neurons of the substantia nigra, is another factor which increasingly recognized as a microglial activator and contributor to neuroinflammation (for details see Section 9). As with neurons, degeneration of astrocytes and microglia impairs nervous system function, making the health of these glial cells equally critical. The aim of this review is not only to summarize the pathological mechanisms underlying neurodegenerative diseases but also to highlight potential therapeutic targets. Here, we first present, for the first time, a concise overview of the principal hypotheses linking glial cells to the early stages of neurodegeneration.

## 2. Hypothesis of Microglial Polarization from the M2 to M1 Phenotype

**Figure 1:**
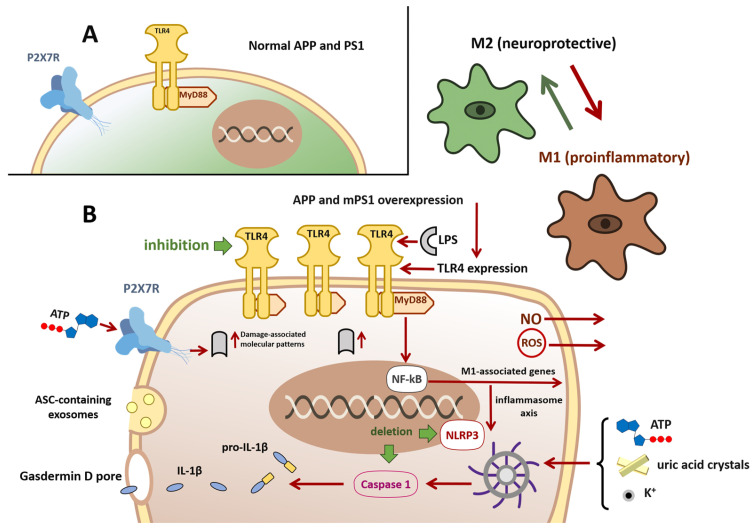
Hypothesis of microglial polarization from the M2 to M1 phenotype and NLRP3 inflammasome activation. Panel (**A**) In the APP/PS1 mouse model of Alzheimer’s disease, microglia initially display an anti-inflammatory, neuroprotective M2 phenotype (green). Disease progression driven by overexpression of mutant human APP and PS1 and by accumulation of β-amyloid causes microglia to shift toward a proinflammatory M1 phenotype (dark brown). Panel (**B**) Pathological M1 microglia exhibit upregulated TLR4 expression. TLR4 activation, for example, by lipopolysaccharide (LPS), initiates a MyD88-dependent signaling cascade that promotes NF-κB nuclear translocation and transcription of M1-associated genes, driving the inflammatory response. NF-κB activation can promote assembly and activation of the (NLRP3) inflammasome, resulting in caspase-1 activation and cleavage of pro-IL-1β and pro-IL-18 to mature IL-1β and IL-18 and their release via gasdermin D pore. Caspase-1 activation also induces gasdermin D-mediated pyroptotic cell death. M1 microglia can propagate inflammasome activation to neighboring cells via exosome release of the ASC adaptor. NLRP3 activation may also be triggered by extracellular ATP, uric acid crystals, elevated extracellular K^+^, or stimulation of P2X7 purinergic receptors. Chronic persistence in the M1 state leads to release of neurotoxic mediators, including reactive oxygen species (ROS) and nitric oxide (NO). Pharmacological inhibition of TLR4 attenuates NF-κB signaling and favors reversion toward the M2 phenotype. Genetic deletion of NLRP3 or caspase-1 reduces cognitive deficits and promotes an M2-biased microglial state. Abbreviations: APP, Amyloid precursor protein; PS1, Presenilin 1; TLR-4, toll-like receptor 4; MyD88, Myeloid differentiation primary response 88; NF-κB, nuclear Factor kap-pa-light-chain-enhancer of activated B cells; NLRP3, NOD-like receptor family pyrin domain containing 3; ILs, Interleukins; ASC, Apoptosis-associated Speck-like protein containing a CARD; P2X7, Purinoceptor 7.

One of the most widely accepted hypotheses in neurodegeneration proposes that microglia undergo a phenotypic switch from an M2 (Figure 1A), neuroprotective state to an M1, proinflammatory state (Figure 1B). This transition is driven by proinflammatory cytokines, including interferon-gamma (IFN-γ), lipopolysaccharide (LPS), and other immune mediators, which tip the balance toward an M1 profile [50] (Figure 1). In the context of neurodegenerative diseases, extensive tissue damage and microbial invasion further amplify signals that favor the M1 phenotype [51].

The resulting exaggerated inflammatory response releases neurotoxic factors such as reactive oxygen species, nitric oxide, and proinflammatory cytokines, which can directly injure neurons and exacerbate cell death [40,47,48,52]. Accordingly, chronically reactive microglia are now recognized as key contributors to both the initiation and progression of neurodegenerative processes [7,12,15,53,54,55].

A central regulator of this phenotypic shift is Toll-like receptor 4 (TLR4), a transmembrane pattern-recognition receptor expressed on microglia that activates the innate immune response. In the APP/PS1 mouse model of Alzheimer’s disease, which overexpresses mutant human amyloid precursor protein (APP) and presenilin 1 (PS1) and accumulates β-amyloid, microglia display increased TLR4 expression compared with wild-type controls [56]. Engagement of TLR4 by LPS or endogenous damage-associated molecular patterns activates the myeloid differentiation primary response 88 (MyD88)-dependent pathway, leading to nuclear factor kappa-light-chain-enhancer of activated B cells (NF-κB) translocation and transcription of M1-associated genes (Figure 1B) [56].

TLR4 is a type I transmembrane receptor with an extracellular leucine rich repeat (LRR) ligand-binding domain, a single transmembrane helix, and a cytoplasmic Toll/IL 1 receptor (TIR) signaling domain. Its activation requires dimerisation and accessory proteins such as myeloid differentiation factor-2 (MD-2) and cluster of differentiation 14 (CD14), which enable binding of LPS or endogenous damage associated molecular patterns. Structural studies show that ligand binding induces conformational changes in the LRR domain, promoting TLR4/MD-2 dimerization and juxtaposition of the TIR do-mains, which then recruit adaptor proteins (MyD88 or TRIF) to trigger NF κB and interferon regulatory factor (IRF) pathways [57].

Experimental inhibition of TLR4 attenuates NF-κB activation and promotes a return toward the M2 phenotype. Lang et al. [58] demonstrated in vitro that blocking LPS-induced TLR4 signaling reduced NF-κB activity and shifted microglia from M1 to M2. Complementary studies have shown that TLR4 blockade suppresses the MyD88/NF-κB/ NOD-like receptor family pyrin domain containing 3 (NLRP3) inflammasome axis, lowering NLRP3 levels and curbing the release of interleukin-1β and other proinflammatory mediators [56], (Figure 1B).

In summary, pathological stimuli in neurodegenerative diseases drive microglial polarization toward a neurotoxic M1 state, with TLR4 serving as a pivotal mediator. Therapeutic strategies aimed at inhibiting TLR4 or its downstream signaling pathways hold promise for preserving microglial homeostasis and mitigating neuronal injury. Nevertheless, because M1 polarization often follows initial tissue damage or infection, effective interventions may also need to address these upstream insults.

In addition to receptor-mediated signaling, the redox environment strongly influences microglial polarization. Enzymes such as superoxide dismutase (SOD), which require copper and zinc cofactors for catalytic activity, play a central role in detoxifying superoxide radicals. Alterations in metal availability or SOD function can exacerbate oxidative stress, thereby favoring NF-κB activation and a shift from the anti-inflammatory M2 phenotype toward the proinflammatory M1 state [59].

## 3. NLRP3 Microglial Activation Hypothesis

NLRP3 is a member of the nucleotide-binding oligomerization domain (NOD), leucine-rich repeat (LRR), and pyrin domain-containing (NLRP) receptor family within the broader NOD-like receptor superfamily. These cytosolic receptors detect pathogen-associated or damage-associated molecular patterns delivered via phagocytosis or membrane pores. In microglia, NLRP3 senses danger signals—such as extracellular adenosine triphosphate (ATP), uric acid crystals, or elevated extracellular K+ from damaged cells—and assembles into the NLRP3 inflammasome, a multiprotein complex composed of the NLRP3 sensor, the ASC adaptor (apoptosis-associated speck-like protein containing a CARD), and the effector protease caspase-1 (ICE) (Figure 1B). Activation of caspase-1 drives the maturation and release of IL-1β and interleukin-18 (IL-18) and triggers pyroptosis, a form of proinflammatory cell death mediated by gasdermin D pores [60]. Beyond its structural assembly, the NLRP3 inflammasome exerts its function through the catalytic activity of caspase-1. Caspase-1 is a cysteine protease whose active site contains a critical cysteine residue that performs nucleophilic attack on peptide bonds, enabling cleavage of pro-IL-1β and pro-IL-18 into their mature, bioactive forms. This cysteine-dependent mechanism is essential for inflammasome-driven cytokine release and pyroptotic cell death, thereby linking structural activation of the NLRP3/ASC/caspase-1 complex to downstream neuroinflammatory processes [52,61,62,63].

In neurodegenerative disease, M1-polarized microglia may amplify NLRP3 activation (Figure 1B). Extracellular ATP engages P2X purinergic receptor 7 (P2X7) on microglia, whose expression rises in late-stage Alzheimer’s disease [64,65]. P2X7 stimulation promotes K+ efflux and NLRP3 assembly. Moreover, microglia release ASC-containing exosomes that propagate inflammasome activation in neighboring cells [66]. In the APP/PS1 Alzheimer’s mouse 196 model, genetic deletion of NLRP3 or caspase-1 reduces cognitive deficits, shifts microglia toward an M2 phenotype, and enhances β-amyloid clearance [67]. Because P2X7 receptors require high (millimolar) ATP concentrations for activation—far above the micromolar levels that activate other P2X subtypes—robust NLRP3-mediated signaling occurs only after extensive membrane [43,68] (Figure 1B). However, it is important to note that P2X7R-expressing microglia can become markedly activated in response to elevated extracellular ATP levels, thereby exacerbating neuroinflammation and contributing to neurotoxicity [69]. Thus, NLRP3 inflammasome engagement is likely a late event in Alzheimer’s pathogenesis. Nonetheless, therapeutically targeting P2X7R or components of the NLRP3/caspase-1 axis holds promise for dampening microglia-driven inflammation and preventing pyroptotic neuronal loss.

## 4. Hypothesis of Crosstalk Among Microglia, Astrocytes, and Neurons

**Figure 2:**
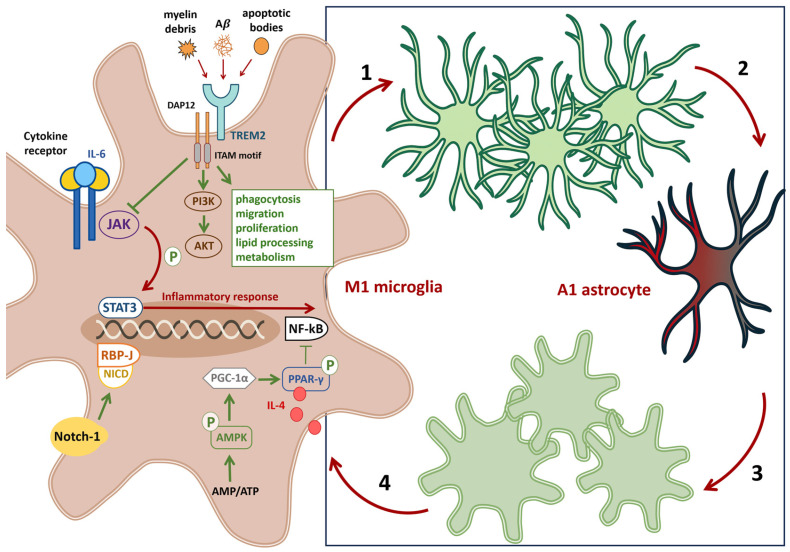
Hypothesis of crosstalk among microglia, astrocytes, and neurons. Panel schematic on the (**right**). An M1 polarized microglial cell releases proinflammatory mediators that act on A2 astrocytes (1), driving their conversion to a neurotoxic A1 phenotype (2) and eliciting a secondary astrocyte-mediated inflammatory response. Proinflammatory A1 astrocytes feed back on neighboring microglia (3), promoting further M1 polarization (4). Pathological strategies on the (**left**). TREM2 signaling. Overexpression or activation of TREM2 (via DAP12) enhances microglial phagocytosis, migration, lipid handling, proliferation, lysosomal degradation, and metabolism, and activates the PI3K–AKT pathway to bias microglia toward the M2 phenotype. Therapeutic strategies on the (**left**). Activation of the Notch1 receptor in microglia induces proteolytic release of the Notch intracellular domain NICD, which translocates to the nucleus and engages RBP-J, favoring retention of a noninflammatory phenotype. Activation of the AMPK/PGC 1α/PPAR γ axis inhibits NF-κB, reducing proinflammatory signaling. Inhibition of the JAK2–STAT1/3 pathway represents an additional anti-inflammatory approach. Blocking proinflammatory cytokine signaling in microglia (for example, antagonism of IL-6 receptors) while augmenting neuroprotective signals such as IL-4 can limit harmful glial cross-activation and promote restoration of homeostatic microglial and astrocytic states. Abbreviations. TREM2, Triggering receptor expressed on myeloid cells 2; DAP12, DNAX-activating protein of 12 kDa; PI3K–AKT, Phosphoinositide 3-kinase—protein kinase B; Notch1, Neurogenic locus notch homolog protein 1; RBP-J, Recombination signal binding protein for immunoglobulin kappa J region; AMPK, Adenosine monophosphate-activated protein kinase; PGC 1α, Peroxisome proliferator activated receptor-gamma coactivator; PPAR γ, Peroxisome proliferator-activated receptor gamma; JAK2, Janus kinase 2; STAT, Signal transducer and activator of transcription.

A series of recent studies highlights the critical role of cross-talk among astrocytes, microglia, and neurons in the pathogenesis of neurodegenerative diseases. According to Li et al. [70], reactive astrocytes and microglia establish a proinflammatory positive feedback loop that dysregulates and amplifies the neuroinflammatory response (Figure 2, 1–4). As noted above, in neurodegenerative diseases, microglia transition to a neurotoxic, M1 (proinflammatory) phenotype at relatively late stages of disease progression. M1-polarized microglia secrete inflammatory mediators that activate A1 astrocytes, eliciting a secondary inflammatory response; mediators released by A1 astrocytes then reinforce M1 polarization of microglia. This self-perpetuating interaction drives chronic astrogliosis and microglial activation within the CNS, culminating in neuronal damage [3,12,37,71,72,73,74].

Microgliosis is a non-specific reactive response characterized by microglial proliferation and hypertrophy. It typically precedes astrogliosis and involves recruitment of border-associated macrophages (BAMs) from the meninges, choroid plexus, and perivascular spaces, together with resident microglia, to sites of injury [75]. Astrogliosis (astrocytosis) is defined by astrocyte proliferation, altered molecular signatures, and morphological changes in response to neuronal injury. Severe astrogliosis leads to glial scar formation that impedes axonal regeneration, increases blood–brain barrier permeability, and permits inflammatory T-cell infiltration into the parenchyma [3,10,72,76]. Reactive astrocytes and microglia also damage oligodendrocytes, impairing axonal myelination and further compromising neural circuitry [73,77,78,79]. Given that microglia respond to local pathological stimuli before astrocytes, promoting microglial polarization toward an M2 (anti-inflammatory) phenotype is a logical first step to restore glial homeostasis. This shift can be induced by (see the summary in Figure 2, left):

- Inhibiting nuclear factor κB (NF-κB), which regulates genes involved in immune and inflammatory responses
- Blocking the janus kinase 2–signal transducer and activator of transcription1/3 (JAK2–STAT1/3) pathway, overexpression of triggering receptor expressed on myeloid cells 2 (TREM2) on microglial membranes recruits the DNAX-activating protein 12 (DAP12) adaptor to suppress JAK2–STAT1/3 signaling while enhancing phagocytosis, migration, lipid processing, proliferation, lysosomal degradation, and metabolism [80,81,82]
- Activating alternative pathways (phosphoinositide 3-kinase—protein kinase (PI3K/AKT), neurogenic locus notch homolog protein (Notch), peroxisome proliferator-activated receptor gamma (PPAR-γ), or adenosine monophosphate-activated protein kinase (AMPK)) to drive M2 polarization [83].

More than 50 cytokines and growth factors—including interferons and interleukins—participate in the JAK/STAT cascade, which governs hematopoiesis, immune homeostasis, tissue repair, inflammation, apoptosis, and adipogenesis [84].

Cytokine signaling in the crosstalk among microglia, astrocytes, and neurons is mediated not only by receptor engagement but also by specific phosphorylation events that propagate intracellular responses. Binding of cytokines such as IL-6 to their receptors activates JAK2, which catalyzes phosphorylation of tyrosine residues within the receptor’s intracellular domain. These phosphotyrosine motifs serve as docking sites for STAT3, which is subsequently phosphorylated on a critical tyrosine residue (Tyr705) and, in some contexts, on serine residues (Ser727) to modulate transcriptional activity. The dual Tyr/Ser phosphorylation of STAT3 regulates its dimerization via the Src homology 2 (SH2) domain, nuclear translocation, and transcription of genes involved in inflammation, gliosis, and neuronal survival. Thus, the chemistry of tyrosine and serine phosphorylation provides a mechanistic link between cytokine receptor activation and the transcriptional programs that shape microglial and astrocytic responses in neurodegeneration [84,85,86].

A notable strategy for modulating pathological microglia is delivering anti-inflammatory cytokines such as interleukin-4 (IL-4) (Figure 2), interleukin-10 (IL-10), and transforming growth factor-β (TGF-β), which:

- IL-4 drives proliferation and activation of microglia;
- IL-10 suppresses proinflammatory cytokine production;
- TGF-β regulates cell proliferation, differentiation, and apoptosis

Inhibition of TNFα and IL-1β in BV2 cells treated with soluble amyloid-β oligomers attenuates neuronal cytotoxicity. In APN/5xFAD mice, elevated TNFα and IL-1β correlate with widespread microglial activation, reduced clustering around fibrillar plaques, and abnormal microglial morphology [40,83,87,88,89,90,91].

Chuang et al. [92] showed that a small molecule protects rat hippocampal neurons from Aβ-induced toxicity and attenuates LPS-driven neuroinflammation by reducing nitric oxide production, downregulating inducible nitric oxide synthase (iNOS) and IL-1β, suppressing IL-6 and TNFα secretion, and blocking NF-κB activation in BV-2 cells.

Resveratrol, a plant-derived polyphenol, activates sirtuin-1 (SIRT1), a nicotinamide adenine dinucleotide–dependent deacetylase that regulates energy metabolism, aging, oxidative-stress responses, and inflammatory pathways, thereby inhibiting microglial M1 transformation [93,94].

The microglial landscape includes multiple disease-associated phenotypes—activated response microglia (ARM), disease-associated microglia (DAM), microglial neurodegenerative phenotype (MGnD), lipid droplet–accumulating microglia (LDAM), white matter–associated microglia (WAM), and dark microglia (DM)—each defined by distinct localization, markers, and functions. A refined classification of these subtypes will inform targeted therapeutic strategies [52,95,96,97].

In summary, bidirectional signaling between microglia and astrocytes critically modulates CNS homeostasis and pathology. While shifting microglia toward an M2 phenotype suppresses inflammation and protects neurons in model systems, its efficacy across diverse phenotypes and its ability to reverse established neurodegeneration before the therapeutic window closes remain open questions.

## 5. The Microglial Dysfunction Hypothesis: Impaired Phagocytosis and Aβ Clearance

This hypothesis posits interactions between beta-amyloid (Aβ) and microglia. Aβ activates microglia via Toll-like receptors (TLRs), triggering the release of neuroinflammatory mediators [30,98,99,100,101].

**Figure 3:**
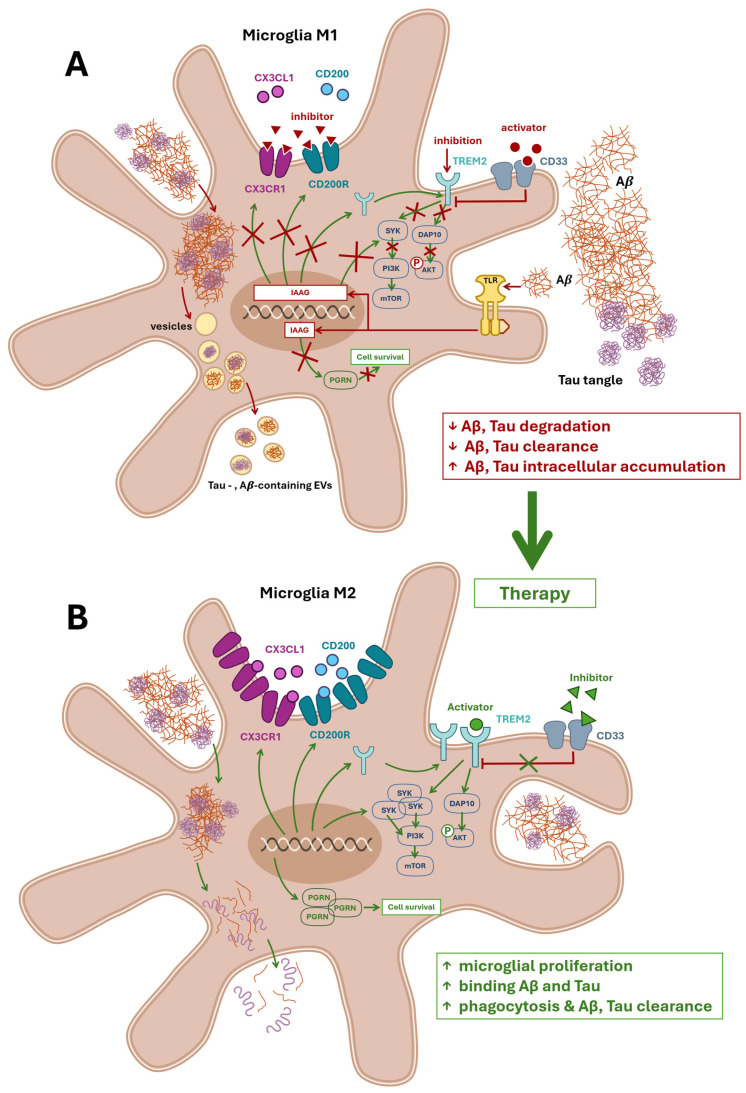
Microglial dysfunction and therapeutic restoration in Alzheimer’s disease. Panel (**A**) illustrates the microglial dysfunction hypothesis, highlighting impaired phagocytosis and reduced clearance of amyloid beta (Aβ) and tau proteins. In Alzheimer’s disease, Aβ activates microglia via Toll-like receptors (TLRs), inducing a proinflammatory M1 phenotype and triggering the release of neuroinflammatory mediators. This response promotes the expression of genes associated with impaired autophagy (IAAG), resulting in the downregulation of key surface receptors—CX3CR1, CD200R, and TREM2—while their activity may be further inhibited by extracellular antagonists. Pathological microglia internalize Aβ and tau but fail to fully degrade them, leading to intracellular accumulation and packaging into extracellular vesicles (EVs). These EVs propagate neurotoxicity and promote the conversion of Aβ into more harmful species. CD33 further suppresses TREM2 signaling, while impaired CX3CL1–CX3CR1 and CD200–CD200R interactions diminish anti-inflammatory regulation. TREM2 signaling via SYK and DAP10 is disrupted, impairing activation of the PI3K–AKT–GSK-3β–mTOR cascade essential for microglial survival, proliferation, and phagocytic function. Reduced expression of progranulin (PGRN) further compromises microglial viability. The red box summarizes these pathological outcomes. Panel (**B**) depicts therapeutic strategies aimed at restoring microglial function. Activation and expression of CX3CR1, CD200R, and TREM2 are enhanced, promoting anti-inflammatory signaling and phagocytic activity. Inhibition of CD33 relieves suppression of TREM2, enabling downstream recruitment of DAP10 and activation of SYK. This reinstates the PI3K–AKT–GSK-3β–mTOR signaling axis, supporting microglial metabolic function and acquisition of the disease-associated microglia (DAM) phenotype. Upregulation of PGRN expression fosters microglial survival and enhances clearance of Aβ and tau. The green box highlights therapeutic outcomes. Abbreviation: TREM2, triggering receptor expressed on myeloid cells 2; CX3CL1, chemokine C-X3-C motif ligand 1; CD200, neuronal membrane glycoprotein OX-2; SYK, spleen tyrosine kinase; DAP10, hematopoietic cell signal transducer; CD33, Siglec-3; PGRN, precursor protein of granulin; mTOR, Mammalian target of rapamycin; PI3K, phosphoinositide 3-kinase; GSK-3β, Peroxisome proliferator-activated receptor gamma coactivator 1-alpha; IAAG, impaired autophagia associated genes; EVs, extracellular vesicles; TLR, Toll-like receptor; CX3CR1, CX3C motif chemokine receptor 1; CD200R, cell surface glycoprotein CD200 receptor 1.

Under physiological conditions, microglia efficiently phagocytose and clear Aβ. In Alzheimer’s disease, however, pathological microglia engulf Aβ and tau but fail to degrade these proteins fully, resulting in their intracellular accumulation (Figure 3A). These aggregates are packaged into microglial extracellular vesicles (EVs), which in turn promote EV release. Furthermore, the lipid constituents of large EVs can facilitate the conversion of Aβ into more neurotoxic species [101,102,103,104,105]. In a mouse model, Aβ plaque–associated microglia exhibited increased secretion of tau-containing EVs, contributing to the propagation of tau pathology in AD [47,101,105,106].

Impaired microglial clearance of amyloid β (Aβ) is strongly influenced by the biochemical properties of Aβ itself. It stems from peptides’ hydrophobic motifs in central/C-terminal regions, promoting β-sheet formation, fibrillization, and rendering aggregates resistant to proteolysis. These structural features facilitate ligand interactions with cell surface receptors and hinder efficient phagocytosis. In addition, metal ions such as zinc can bind to histidine residues within Aβ, stabilizing fibrillar assemblies and further reducing solubility. Serine protease-mediated degradation (e.g., neprilysin, insulin-degrading enzyme) fails against fibrillar Aβ, leading to impaired proteolytic clearance. Together, the interplay of hydrophobicity, β sheet structural motifs, and metal coordination chemistry contributes to microglial dysfunction and persistence of neurotoxic Aβ deposits [107,108].

The chemokine C-X3-C motif ligand 1 (CX3CL1), expressed by neurons and glial cells, is a proposed target for modulating microglial phagocytic, degradative, and clearance functions. Like other chemokines, CX3CL1 orchestrates chemotaxis and the migration of diverse cell populations, including microglia [109,110]. Another potential target is the neuronal membrane glycoprotein OX-2 (CD200). Both CX3CL1 and CD200 suppress the proinflammatory M1 microglial response via their respective surface receptors. In neurodegenerative diseases, the function of these receptors is compromised, and levels of CD200 and CD200 receptor 1 (CD200R)—and the strength of their interaction—are markedly reduced [3,73,111,112] (Figure 3A).

Impaired fractalkine signaling via the CX3CL1– CX3C motif chemokine receptor 1 (CX3CR1) axis may promote the emergence of disease-associated microglia (DAM), a distinct microglial phenotype [95], yet consensus regarding CX3CR1 is lacking. In 5xFAD mice, Puntambekar et al. [113] demonstrated that CX3CR1 deficiency impairs clearance of fibrillar Aβ, leading to accumulation of neurotoxic Aβ oligomers, neuronal loss, cognitive deficits, and activation of a neurodegenerative microglial phenotype [114,115]. Alternative findings indicate that CX3CR1 deficiency enhances microglia-mediated Aβ clearance but also triggers microglial hyperactivation, elevates IL-6 and IL-1α secretion, and impairs memory in APP/PS1 transgenic mice. In contrast, Cho et al. [114] and Pawelec et al. [112] demonstrated that CX3CR1 deficiency prevents neuronal loss in the 3xTg-AD mouse model. CX3CR1 has also been implicated in promoting tau protein clearance [116].

Another promising therapeutic target is the microglial receptor TREM2, which governs transitions among distinct microglial phenotypes (Figure 3B) [38,117]. In neurodegenerative conditions, TREM2 expression is elevated in both DAM and microglial neurodegenerative (MGnD) phenotypes, and some studies have described a distinct TREM2-high microglia subtype [52,95,97]. TREM2 overexpression in a mouse model of vascular dementia improves spatial memory performance and reduces neuronal loss. It suppresses microglial M1 polarization by decreasing iNOS expression and proinflammatory cytokine secretion while promoting M2 polarization and elevating anti-inflammatory cytokine levels. In the 5XFAD mouse model, chronic TREM2 activation enhances microglial recruitment to amyloid plaques, diminishes amyloid deposition, and ameliorates memory [118,119,120]. In the APP/PS1 model, increasing TREM2 levels reduces synaptic and neuronal loss and upregulates molecular markers of the M2 phenotype [73,81,120,121,122,123]. TREM2 signals via the spleen tyrosine kinase (SYK) pathway and the hematopoietic cell signal transducer (HCST, also known as DNAX-activating protein 10 (DAP10)) pathway [124] (Figure 3A,B). SYK plays a central role in adaptive immune receptor signaling and participates in diverse regulatory processes [125]. DAP10 also contributes to immune regulation [126,127]. DAP10 deficiency alters phosphorylation of protein kinase B (AKT) and glycogen synthase kinase-3β (GSK-3β), impairing microglial proliferation, survival, and phagocytic activity (Figure 3B). SYK-deficient microglia fail to phagocytose Aβ plaques, accelerating amyloid pathology. Moreover, SYK loss disrupts the PI3K–AKT– peroxisome proliferator-activated receptor gamma coactivator 1-alpha (GSK-3β)–mammalian target of rapamycin (mTOR) signaling cascade required for microglia to acquire the DAM phenotype and bind Aβ proteins [124]. Conversely, one study proposes that suppressing TREM2 activation in neurodegenerative disease may be protective, since TREM2 signaling through the TREM2–Apolipoprotein E (ApoE) axis drives microglia toward the MGnD phenotype and promotes neuronal loss [90,128] (Figure 3A).

Another potential target for modulating microglial function is the transmembrane receptor CD33 (siglec-3), expressed on myeloid-lineage cells and functionally linked to TREM2. In AD models, CD33 inhibition has yielded beneficial effects [129,130,131]. For instance, CD33 knockout in 5xFAD mice improved cognition and reduced Aβ burden; these effects were abrogated by concurrent TREM2 knockout [131]. A combined strategy of CD33 inhibition and TREM2 activation may represent an optimal therapeutic approach [132] (Figure 3B). Reports reveal an isoform-dependent dual role for CD33. In 3BV2 mouse microglial cells, elevated expression of the long isoform hCD33M correlates with reduced phagocytic activity. In contrast, predominance of the short isoform hCD33m enhances phagocytosis and migration while diminishing cell adhesion [83].

Progranulin (PGRN) is a relatively under-investigated yet highly promising target for microglial modulation. As the precursor protein of granulin (GRN), PGRN is expressed by both microglia and neurons, where it promotes neuronal survival, maintains lysosomal homeostasis, and regulates inflammatory processes. Progranulin deficiency causes neuronal ceroid lipofuscinosis—characterized by pathological accumulation of toxic lipofuscin pigments that impair lysosomal function—and frontotemporal dementia, and is associated with an increased risk of AD and other neurodegenerative diseases [133,134]. In a transgenic mouse model of AD, PGRN overexpression inhibited A*β* deposition, reduced cerebral amyloid burden, and prevented spatial memory deficits and hippocampal neuronal loss [135], (Figure 3A,B).

Although the literature reports conflicting findings regarding these targets [52,83], there have also been clear successes in restoring microglial function and modulating phenotypes. These discrepancies may arise from differences in molecular forms of the same proteins (e.g., soluble versus insoluble) and variations in disease stage (e.g., pre- versus post-microgliosis) [52,83,95,97]. It is essential to consider combined influences: for example, optimal interventions may involve activating TREM2 while concurrently restoring SYK function and inhibiting CD33. Modulation of TREM2 impacts the regulatory kinase GSK-3*β*, which has a distinct role in the pathogenesis of various neurodegenerative diseases [136], and is associated with the PI3K–AKT–GSK-3*β*–mTOR signaling axis, a critical pathway for cellular metabolism, growth, and proliferation (Figure 3A,B).

Effective microglial targeting requires not only restoring homeostatic function or skewing toward the M2 phenotype but also maintaining the balance between non-pathological M1 and M2 states, which is critical in neurodegenerative diseases [12,24,137]. This balance can be modulated via the P2X purinergic receptor 7 (P2X7R), a member of the ATP purinoceptor family expressed on microglia. Transient elevations in extracellular ATP activate P2X7R, trigger autophagy, and promote an M1/M2 hybrid phenotype. Maintaining a dynamic equilibrium between M1 and M2 states in microglia is essential for DAM emergence [83].

## 6. Astrocytic Scar Formation Hypothesis

The hypothesis that astrogliosis, induced by neurodegenerative processes, leads to glial scar formation is fundamentally distinct from previously discussed models. Although scar formation is more commonly associated with peripheral tissues, emerging evidence suggests that similar processes may occur in the central nervous system (CNS) following chronic cellular and tissue injury. This hypothesis posits that glial scars may impair neuronal regeneration by obstructing axonal growth post-injury, while simultaneously contributing to tissue repair and structural stabilization [138,139,140,141].

**Figure 4:**
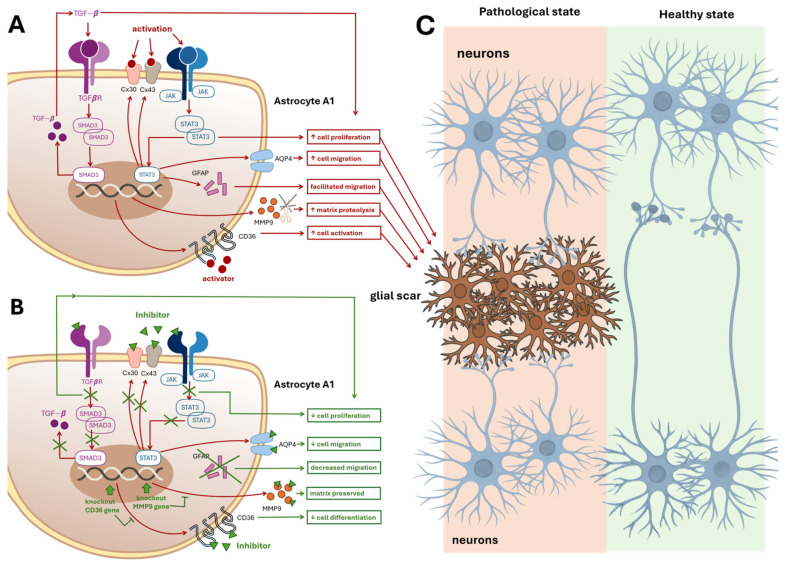
Astrocytic Scar Formation and Therapeutic Modulation. Panel (**A**): This panel illustrates the intracellular and extracellular cascades within proinflammatory A1-type astrocytes that contribute to glial scar formation, which impairs neuronal recovery and axonal outgrowth. Key molecular contributors include: (i) Aquaporin-4 (AQP4) is a protein that maintains osmotic balance and facilitates astrocyte migration. (ii) Matrix Metalloproteinase-9 (MMP-9) degrades extracellular matrix, promoting motility and matrix remodeling. (iii) Intermediate Filament Proteins (GFAP): Support cytoskeletal dynamics and directed migration. (iv)Connexins (Cx30 and Cx43): Transmembrane proteins forming gap junctions that enhance astrocyte activation and intercellular signaling. (v) JAK–STAT3 Signaling Pathway: Promotes astrocyte proliferation and migration, STAT3 regulates transcription of GFAP, Cx43, and AQP4. (vi) SMAD3, a TGF-β pathway transducer, and CD36, a membrane protein, both contribute to proliferation and scarring. SMAD3 knockout reduces scar formation. Each molecular factor is linked via red arrows to functional outcomes: cell migration, proteolysis, activation, and proliferation, represented in red boxes. These processes converge toward Panel C, indicating their collective role in scar development. Panel (**B**): Therapeutic Inhibition of Scar-Forming Pathways. This panel depicts an A1 astrocyte in which key scar-promoting molecules are therapeutically inhibited. Blockade of AQP4, Cx30, Cx43, CD36, MMP-9, GFAP, SMAD3, and STAT3—either directly or via transcriptional suppression—leads to: (i) Decreased cell migration, (ii) Decreased cell proliferation, (iii) Preserved extracellular matrix. These outcomes are shown in green boxes, representing potential therapeutic strategies to prevent glial scar formation and promote CNS recovery. Panel (**C**): Impact of Glial Scar on Neuronal Connectivity This panel contrasts two tissue states: **Left side**: Pathological tissue with a glial scar in the center, disrupting neurite extension between neurons positioned above and below. Red arrows from Panel A’s red boxes point to the scar, indicating its molecular origins. **Right side**: Healthy tissue without a glial scar, allowing uninterrupted neuronal connectivity and synaptic regeneration. Abbreviations: AQP4, Aquaporin-4; MMP-9, Matrix metalloproteinase-9; GFAP, Glial fibrillary acidic protein; Cx30, Connexin 30; Cx43, Connexin 43; JAK, Janus kinase; STAT3, Signal transducer and activator of transcription 3; TGF-β, Transforming growth factor-β; CD36, Cluster of differentiation 36; SMAD3, Mothers against decapentaplegic homolog 3.

Astrocyte migration toward the injury site plays a key role in scar formation and can be modulated by several molecular pathways (Figure 4). Aquaporin-4 (AQP4), a water channel protein expressed in astrocyte membranes, facilitates migration by maintaining water–electrolyte balance and osmotic pressure (Figure 4A). Matrix metalloproteinase-9 (MMP-9), a zinc-dependent enzyme involved in extracellular matrix degradation, also regulates astrocyte motility. In murine models, both MMP-9 inhibition and AQP4 knockout significantly reduce astrocyte migration (Figure 4B) [142,143].

Additional contributors to glial scarring include type III intermediate filament proteins such as vimentin and glial fibrillary acidic protein (GFAP). Dual knockout of these proteins in mice results in diminished post-traumatic glial scar formation [143]. Con-nexins—transmembrane proteins that form intercellular gap junctions—also represent potential therapeutic targets. Astrocytes express high levels of connexin 30 (Cx30) and connexin 43 (Cx43), although their expression is downregulated in reactive astrocytes [143]. The JAK–STAT3 signaling pathway, previously discussed, promotes astrocyte proliferation at sites of injury (Figure 4). Inhibition of STAT3 via JAK signaling has been shown to suppress both proliferation and migration of reactive astrocytes following spinal cord injury, suggesting a similar mechanism may operate elsewhere in the CNS (Figure 4B, C). Notably, STAT3 regulates the transcription of GFAP, Cx43, and AQP genes.

Other promising molecular targets include mothers against decapentaplegic homolog 3 (SMAD3), a signal transducer in the transforming growth factor-*β* (TGF-*β*) pathway, and cluster of differentiation 36 (CD36), an integral membrane protein expressed on various cell types. SMAD3 knockout significantly reduces glial scar formation [142,143], while CD36 deficiency in stroke model mice attenuates astrocyte proliferation and delays wound-gap closure. CD36 knockout mice also exhibit reduced scar formation [144] (Figure 4B,C).

Despite these findings, the glial scar hypothesis remains contentious. Some researchers argue that glial scars may exert neuroprotective effects by limiting the spread of damage and inflammation [145]. Moreover, many current approaches focus on suppressing reactive astrocyte activity rather than restoring astrocytes to a physiologically normal state. Therapeutic strategies aimed at normalization may offer more effective mitigation of scarring-related damage.

Recent studies in Acomys (spiny mouse), a species with exceptional regenerative capacity, support this notion. These rodents are capable of regenerating spinal cord fibers without forming astrocytic scars at the injury site during recovery [146,147].

## 7. Cellular Reprogramming

In recent years, research has increasingly focused on reprogramming astrocytes and other glial cells into mature, functional neurons to replenish lost neuronal populations, rather than merely targeting their pathological phenotypes. For instance, Yin and coauthors [148] demonstrated that human cortical astrocytes reprogrammed in vitro remained viable for over seven months and exhibited robust functionality, including synaptic burst activity. Furthermore, neurons derived from astrocytes have been shown to form synaptic connections with endogenous cortical neurons in adult mice in vivo [149].

Guo and coauthors [150] reported that reactive astroglia—following brain injury or in Alzheimer’s disease—can be reprogrammed into functional glutamatergic neurons. Their study also showed that NG2 glia can be converted into both glutamatergic and GABAergic neurons via expression of the neuronal transcription factor NeuroD1. The overarching goal is to restore functional neuronal populations lost due to injury or disease. NG2 cells, also known as polydendrocytes, typically serve as precursors to oligodendrocytes. However, evidence suggests that they can differentiate into proliferating reactive astrocytes within glial scars [143].

NG2 glia have demonstrated neuroprotective properties in prion-infected mouse models, where their depletion exacerbates neurodegeneration and accelerates prion pathology [151]. Additionally, both NG2 glial cell density and differentiation capacity are reduced in Alzheimer’s disease patients [152], with similar declines observed in APP23 transgenic mice [153].

To date, a substantial body of research has explored astrocyte reprogramming in the context of neurodegenerative diseases and other pathological conditions. This approach holds promise not only for neuronal replacement but also for attenuating proinflammatory astroglia phenotypes, offering a dual therapeutic benefit in the treatment of various neurodegenerative disorders [154,155,156].

## 8. The Mitochondrial Hypothesis of Microglial and Astrocyte Dysfunction

Neurodegenerative diseases (NDDs) disrupt microglial mitochondrial function, potentially acting as an early trigger for pathological microglial activation. Abnormalities in mitochondrial DNA (mtDNA), impaired bioenergetics, and defects in the mitochondrial quality-control machinery all contribute to this dysfunction [157].

**Figure 5:**
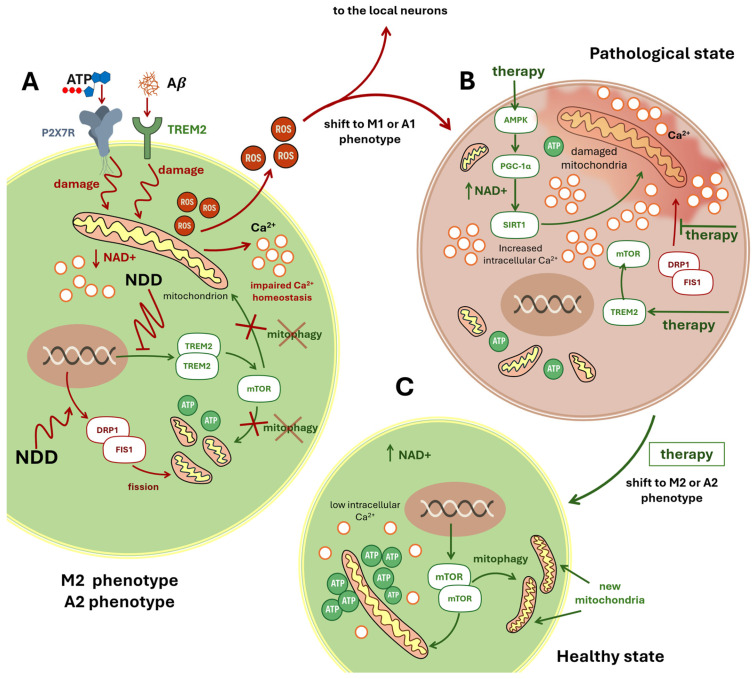
The Mitochondrial Hypothesis of Neurodegeneration. This schematic illustration presents the mitochondrial hypothesis of neurodegeneration, using microglial cells as a representative model. Panel (**A**): Initiation of Mitochondrial Damage in M2 Microglia. Microglia in the M2 phenotype are shown under early neurodegenerative conditions. Extracellular ATP activates the purinergic receptor P2X7R, while extracellular amyloid-β (Aβ) engages TREM2. These signals converge to damage intracellular mitochondria (depicted with red zigzag arrows), triggering the release of reactive oxygen species (ROS) and calcium ions (Ca^2+^). Elevated intracellular Ca^2+^ levels and persistent stress activate the DRP1–FIS1 pathway, promoting excessive mitochondrial fission. Fragmented mitochondria exhibit reduced ATP production. Concurrently, NDD suppresses the TREM2–mTOR axis, impairing mitophagy—the selective clearance of damaged mitochondria—illustrated by a red cross over the term “mitophagy”. Panel (**B**): Pathological Shift to M1 Phenotype and Therapeutic Targets. A red arrow indicates the transition to the M1 proinflammatory phenotype. The microglial cell displays fragmented mitochondria, elevated Ca^2+^, and a reddish background symbolizing inflammation. Potential therapeutic strategies are highlighted in green: (i) Activation of the AMPK–PGC-1α–SIRT1 pathway enhances NAD levels and supports mitochondrial recovery (NAD^+^: arrow up corresponds to raise). (ii) Restoration of the TREM2–mTOR pathway may reinstate mitophagy. (iii) Inhibition of the DRP1–FIS1 axis aims to reduce excessive mitochondrial fission. Panel (**C**): Restoration to Healthy M2 State. A green arrow from Panel B leads to Panel C, labeled “Healthy State,” with the accompanying text “shift to M2 phenotype.” Here, microglia exhibit restored mitochondrial integrity, reduced intracellular Ca^2+^, and elevated ATP production. Reactivated mTOR signaling promotes mitophagy, resulting in the emergence of healthy, newly formed mitochondria and a return to the neuroprotective M2 phenotype. Abbreviations: ROS, Reactive oxygen species; TREM2, Triggering receptor expressed on myeloid cells 2; DRP1, Dynamin-related protein 1; FIS1, Mitochondrial fission protein 1; mTOR, Mammalian target of rapamycin; AMPK, AMP-activated protein kinase; PGC-1α, Peroxisome prolifera-tor-activated receptor gamma coactivator 1-alpha; NAD^+^, Nicotinamide adenine dinucleotide; SIRT1, Sirtuin 1; ATP, Adenosine triphosphate; Aβ, Amyloid-β; Ca^2+^, Calcium ion.

Interactions between amyloid-*β* (A*β*) and the TREM2 and P2X7 receptors further exacerbate microglial mitochondrial damage (Figure 5A) [158]. In 5×FAD mice lacking TREM2, mTOR signaling—which governs autophagy—was impaired, leading to reduced mitochondrial mass and lower ATP levels in microglia. This implicates TREM2 deficiency as a critical driver of microglial mitochondrial dysfunction [158].

Accumulation of damaged mitochondria elevates reactive oxygen species (ROS) production, which in turn inflicts oxidative damage on neighboring neural and glial cells [159,160,161].

Activation of the AMP-activated protein kinase (AMPK) pathway shifts microglia toward an anti-inflammatory M2 phenotype, stimulates PGC-1α, and increases nicotinamide adenine dinucleotide (NAD) levels (Figure 5B,C). Elevated NAD activates the deacetylase Sirtuin 1 (Sirt1), collectively promoting mitochondrial biogenesis, enhancing oxidative phosphorylation, and facilitating mitophagy [4,83] (Figure 5B,C).

An alternative mechanism of microglial mitochondrial deficiency is excessive Drp1/Fis1-mediated fission. Driven by dynamin-related protein 1 (Drp1) and mitochondrial fission protein 1 (Fis1), this pathological fission fragments mitochondria, triggering innate immune signaling, inducing A1-phenotype astrocytes, and culminating in neuronal apoptosis [162].

Astrocytes also exhibit profound mitochondrial dysfunction in NDDs. Uptake of Aβ and α-synuclein aggregates impairs their respiratory capacity [163]. In ischemia models, a loss of healthy mitochondria in astrocytic processes correlates with neuronal death, while mitochondrial damage activates astrocytes to release proinflammatory cytokines and disrupt Ca^2+^ homeostasis (Figure 5). During ischemic injury, astrocytes can transfer healthy mitochondria to neurons, but if dysfunctional mitochondria are transferred—as may occur in NDDs—neuronal viability is compromised [164]. In brains of 18-month-old mice, astrocytes show pronounced mitochondrial fragmentation accompanied by a tenfold upregulation of Drp1 [165].

Mitochondrial defects in astrocytes also impair oxidative phosphorylation, essential for fatty-acid oxidation and lipid homeostasis. Such defects contribute to synaptic loss, neuroinflammation, cognitive decline, and reactive astrogliosis [166].

The interaction between the extensive endoplasmic reticulum (ER) network of astrocytes and mitochondria plays a particularly important role in astrocytic function. Regions of close apposition and membrane contact between the ER and mitochondria (mitochondria-associated membranes, MAMs) maintain precise coordination between these organelles and likely determine ATP production, synthesis of proteins and membrane components, stress responses, induction of mitophagy, and initiation of apoptosis. A marked reduction in glucose metabolism in astrocytes is already observed at early stages of neurodegenerative diseases. With extracellular accumulation of Aβ aggregates, astrocytes adopt a reactive phenotype that, to some extent, initially counteracts neurodegeneration. However, the emergence of proinflammatory phenotypes at more advanced disease stages accelerates, rather than delays, the progression of dementia. In particular, ATP deficiency and increased reactive oxygen species (ROS) production contribute to this process (Figure 5A). Studies have also reported ER stress and impaired protein synthesis in glial cells [167,168]. Under these conditions, cytosolic Ca^2+^ levels are elevated while mitochondrial Ca^2+^ is reduced (Figure 5A). Although the precise mechanism remains incompletely understood, evidence suggests that abnormally enhanced ER–mitochondria interactions—resulting from shortened intermembrane distances and elongation of the MAM—may underlie these changes. Dematteis and colleagues have proposed that efficient Ca^2+^ transfer from the ER to mitochondria requires an interorganelle distance of approximately 15–25 nm, whereas in proinflammatory astrocytes this distance is reduced to 10 nm or less [169]. This mechanism could account for both cytosolic Ca^2+^ elevation and mitochondrial Ca^2+^ depletion, leading to an uncompensated bioenergetic deficit.

Although the precise molecular cascades remain to be fully elucidated, converging evidence indicates that early mitochondrial dysfunction in both microglia and astrocytes represents a pivotal risk factor for the onset and progression of neurodegenerative diseases.

## 9. Other Hypotheses

The connection between glial cells and neurodegenerative pathology is broader than the mechanisms discussed in detail here. Our focus has been on glial dysfunction as a primary driver, with neuronal effects mediated through glia. However, several additional processes directly impact neurons and synaptic transmission, with only secondary involvement of glia. While not the central scope of this review, they warrant brief mention.

Impact of redox-active metals. A key mechanism implicated in astrocyte and microglia activation during neurodegeneration is the disruption of metal homeostasis, particularly involving iron and copper. Redox-active Fe^2+^ and Cu^+^ ions can catalyze Fenton-type reactions, generating hydroxyl radicals that drive oxidative stress and lipid peroxidation. These processes contribute to regulated cell death pathways such as ferroptosis and cuproptosis, which impair mitochondrial integrity and energy metabolism. The ensuing redox imbalance activates transcriptional regulators including NF-κB, thereby amplifying the production of proinflammatory mediators such as nitric oxide (NO) and tumor necrosis factor-α (TNF-α). Sustained release of these mediators compromises the neuroprotective functions of glial cells, fostering a cycle of chronic neuroinflammation and neuronal injury. Thus, metal-catalyzed oxidative reactions represent a critical intersection between glial dysfunction, oxidative stress, and progressive neurodegeneration [170,171,172,173].

Neuromelanin accumulation. Neuromelanin, a dark pigment that progressively accumulates with age in dopaminergic neurons of the substantia nigra, has been increasingly recognized as a key factor in neuroinflammation and neurodegeneration. Beyond serving as a byproduct of catecholamine metabolism, neuromelanin can act as a microglial activator: when released from degenerating neurons, neuromelanin granules are phagocytosed by microglia, triggering proinflammatory responses and the release of cytokines and reactive oxygen species. This process contributes to a self-perpetuating cycle of neuroinflammation that exacerbates neuronal vulnerability. Such mechanisms are under active investigation as potential contributors to Parkinson’s disease pathogenesis [174,175]. Recent studies further suggest that neuromelanin may serve as a nidus for α-synuclein aggregation and synucleinopathy, linking pigment accumulation to protein misfolding and progressive dopaminergic cell loss [176,177]. Collectively, these findings highlight neuromelanin as both a biomarker of aging and a potential driver of microglia-mediated neuroinflammatory cascades in Parkinson’s disease.

Ammonia detoxification. Astrocytes normally detoxify ammonia via glutamine synthetase (GS). In Alzheimer’s disease (AD), GS activity is reduced, and hyperammonemia has been observed in both brain and blood. Elevated ammonia promotes Aβ42 accumulation within astrocytes, creating a vicious cycle: Aβ degradation generates ammonia, which fuels the astrocytic urea cycle but also produces excess gamma-aminobutyric acid (GABA) and hydrogen peroxide, impairing synaptic function and memory [178].

Potassium homeostasis. Astrocytes maintain extracellular K^+^ levels through inwardly rectifying potassium channel 4.1 (Kir4.1) channels. In AD, Kir4.1 expression and conductivity decline, particularly in the hippocampus, disrupting K^+^ clearance and impairing neuronal excitability and memory [179].

Zinc and copper regulation. Glial cells regulate synaptic zinc via transporters (ZnTs, ZIPs) and contribute to copper balance. In AD, dysregulated zinc transport elevates extracellular zinc, accelerating Aβ aggregation into neurotoxic fibrils. High zinc and copper levels within amyloid plaques and impaired synaptic zinc/copper homeostasis compromise plasticity and cognition [180].

## 10. Conclusions

In this review, we have summarized the pathological mechanisms underlying several neurodegenerative diseases and highlighted potential therapeutic targets that warrant further attention. Importantly, we provided, for the first time, a synthesis of the principal hypotheses linking glial cells to the early stages of neurodegeneration. Key limitations to consider include protein function being highly dependent on isoform, the existence of multiple microglial phenotypes for which the same therapeutic approaches may not be appropriate, and the need not only to shift phenotypes from M1/A1 to M2/A2 but also to maintain an appropriate balance between them.

Astrocytes and microglia are indispensable for neuronal support and protection, yet early dysfunction in these glial cells often initiates neurodegeneration. Their morphofunctional changes—microglial polarization, inflammasome activation, impaired phagocytosis, astrocytic scar formation, and mitochondrial stress—emerge before clinical symptoms and may drive pathology.

Mitochondrial deterioration in glia is a consistent early hallmark, underscoring the potential of therapies that restore mitochondrial health prior to irreversible neuronal loss. Likewise, the microglial shift from anti-inflammatory M2 to proinflammatory M1 states amplifies neuroinflammation. Preventing this transition through modulation of TLR4, JAK–STAT, NF-κB, cytokine signaling, or TREM2 pathways represents a promising strategy.

Unchecked M1 activation also promotes NLRP3 inflammasome assembly and induces neurotoxic A1 astrocytes, highlighting maladaptive glial–glial interactions as therapeutic targets. Restoring microglial clearance functions via CX3CL1/CX3CR1, CD200–CD200R, TREM2, CD33 inhibition, or progranulin may further mitigate disease progression. Balancing M1/M2 states through P2X7 signaling could help prevent chronic inflammation.

Astrocyte-directed therapies aim to limit scar formation (via AQP4, MMP-9, vimentin/GFAP, connexins) or reprogram reactive glia into neurons. Enhancing mitophagy and suppressing mitochondrial fission also show promise. Given the heterogeneity of glial phenotypes and targets, multitargeted approaches applied at the earliest stages are most likely to succeed.

Recent discoveries reveal astrocytes’ roles in memory and microglia’s involvement in forgetting and fear [181,182,183,184], emphasizing the need for continued investigation of glial biology. Early-phase insights may yield strategies to intercept neurodegeneration before it becomes irreversible.

## 11. Suggestions and Limitations

Despite the encouraging therapeutic avenues outlined, several challenges remain. Many proposed molecular targets are pleiotropic, raising concerns about off-target effects and systemic toxicity. Some of the hypothetical activators, agonists, and antagonists we mentioned have not yet been developed or lack sufficient safety and specificity. We hope this review will draw the attention of researchers to them. The heterogeneity of glial phenotypes across brain regions and disease stages complicates the translation of preclinical findings into clinical therapies. Furthermore, most current studies rely on animal models that only partially recapitulate human neurodegenerative pathology, limiting predictive validity. Future work should prioritize the development of humanized models, longitudinal studies to capture dynamic glial changes, and combinatorial approaches that integrate mitochondrial protection, immune modulation, and synaptic support. Ultimately, therapeutic strategies must balance efficacy with safety, ensuring that modulation of glial activity does not disrupt their essential homeostatic functions.

## Figures and Tables

**Table 1:** Brain-Related Proinflammatory Cytokines.

Proinflammatory Cytokine	CNS Origin (Primary Producers)	General Effects on Neurons
Interleukin-1 beta (IL-1β)	Microglia, Astrocytes, Endothelial cells	Promotes neuronal death and damage (neurodegeneration), synaptic loss
Tumor Necrosis Factor-alpha (TNF-α)	Microglia, Astrocytes, Neurons	Can be pro-apoptotic (inducing cell death) or prevent apoptosis; linked to synaptic excitotoxicity
Interleukin-6 (IL-6)	Microglia, Astrocytes, Endothelial cells	May rescue damaged neurons, preventing synaptic loss, but chronic overexpression disrupts the blood–brain barrier (BBB)
Interleukin-18 (IL-18)	Activated microglia, Astrocytes, Ependymal cells	Pro-apoptotic (inducing cell death)
Interferon-gamma (IFN-γ)	Infiltrating immune cells; induced in the CNS by injury/infection	Associated with enhanced neurogenesis; linked to demyelination.

## Data Availability

No new data were created or analyzed in this study. Data sharing is not applicable to this article.

## Abbreviations

The following abbreviations are used in this manuscript:

Aβ	β-amyloid
AD	Alzheimer’s disease
AKT	Protein kinase B
AMPK	Adenosine monophosphate-activated protein kinase
ApoE	Apolipoprotein E
APP	Amyloid Precursor Protein
AQP4	Aquaporin-4
ARM	Activated response microglia
ASC	Apoptosis-associated Speck-like protein containing a CARD
ATP	Adenosine triphosphate
BAMs	Border-associated macrophages
BBB	Blood–brain barrier
BDNF	Brain-derived neurotrophic factor
CD14	Cluster of Differentiation 14
CD33	Siglec-3
CD36	Cluster of differentiation 36
CD200	Cluster of Differentiation 200
CD200R	Cluster of Differentiation 200 receptor 1
CNS	Central nervous system
Cx30	Connexin 30
Cx43	Connexin 43
CX3CL1	Chemokine C-X3-C motif ligand 1
CX3CR1	CX3C motif chemokine receptor 1
DAM	Disease-associated microglia
DAMPs	Damage-associated molecular patterns
DAP10	DNAX-activating protein 10
DAP12	DNAX-activating protein 12
DM	Dark microglia
DRP1	Dynamin-related protein 1
ER	Endoplasmic reticulum
EVs	Extracellular vesicles
Fe^2+^	Iron
FGF	Fibroblast growth factor
FIS1	Mitochondrial fission protein 1
GABA	Gamma-aminobutyric acid
GFAP	Glial fibrillary acidic protein
GS	Glutamine synthetase
GSK-3β	Peroxisome proliferator-activated receptor gamma coactivator 1-alpha
IAAG	Impaired autophagia associated genes
ICE	Effector protease caspase-1
IFN-γ	Interferon-gamma
Kir4.1	Inwardly rectifying potassium channel 4.1
ILs	Interleukins
IL-1α	Interleukin-1α
IL-1β	Interleukin-1β
IL-4	Interleukin-4
IL-6	Interleukin-6
IL-10	Interleukin-10
IL-18	Interleukin-18
iNOS	Inducible nitric oxide synthase
IRF	Interferon Regulatory Factor
JAK	Janus kinase
JAK2	Janus kinase 2
LDAM	Lipid droplet–accumulating microglia
LPS	Lipopolysaccharide
LRR	Leucine-rich repeat
MAMs	Mitochondria-associated membranes
MD-2	Myeloid differentiation factor-2
MGnD	Microglial neurodegenerative phenotype
MMP-9	Matrix metalloproteinase-9
mTOR	Mammalian target of rapamycin
MyD88	Myeloid differentiation primary response 88
NAD^+^	Nicotinamide adenine dinucleotide
NDD	Neurodegenerative disease
NeuroD1	Neuronal differentiation 1
NF-κB	Nuclear Factor kappa-light-chain-enhancer of activated B cells
NG2	Neuron-glial antigen 2
NICD	Notch intracellular domain
NLRP3	NOD-like receptor family pyrin domain containing 3
NO	Nitric oxide
NOD	Nucleotide-binding oligomerization domain
Notch1	Neurogenic locus notch homolog protein 1
PAMPs	Pathogen-associated molecular patterns
PGC-1α	Peroxisome proliferator-activated receptor gamma coactivator 1-alpha
PGRN	Precursor protein of granulin
PEDF	Pigment epithelium–derived factor
PI3K	Phosphoinositide 3-kinase
PNS	Peripheral nervous system
PPAR-γ	Peroxisome proliferator-activated receptor gamma
PS1	Presenilin 1
P2X7	P2X purinergic receptor 7
RBP-J	Recombination signal binding protein for immunoglobulin kappa J region
ROS	Reactive oxygen species
Ser	Serine
Ser727	Serine-727
SH2	Src homology 2
SIRT1	Sirtuin 1
SMAD3	Mothers against decapentaplegic homolog 3
SOD	Superoxide dismutase
STAT	Signal transducer and activator of transcription
SYK	Spleen tyrosine kinase
TGF-β	Transforming growth factor-β
TIR	Toll/IL 1 receptor
TLR4	Toll-like receptor 4
TNF- α	Tumor necrosis factor α
TREM2	Triggering receptor expressed on myeloid cells 2
TRIF	TIR-domain-containing adapter-inducing interferon-β
Tyr	Tyrosine
Tyr705	Tyrosine-705
VEGF	Vascular endothelial growth factor
WAM	White matter–associated microglia
ZIP	Zrt- and Irt-like protein
ZnTs	Zinc transporters

## References

[1] Von Bartheld C.S., Bahney J., Herculano-Houzel S. (2016). The Search for True Numbers of Neurons and Glial Cells in the Human Brain: A Review of 150 Years of Cell Counting. J. Comp. Neurol..

[2] von Bartheld C.S. (2018). Myths and Truths about the Cellular Composition of the Human Brain: A Review of Influential Concepts. J. Chem. Neuroanat..

[3] D’Egidio F., Castelli V., d’Angelo M., Ammannito F., Quintiliani M., Cimini A. (2024). Brain Incoming Call from Glia during Neuroinflammation: Roles of Extracellular Vesicles. Neurobiol. Dis..

[4] Hou Y., Dan X., Babbar M., Wei Y., Hasselbalch S.G., Croteau D.L., Bohr V.A. (2019). Ageing as a Risk Factor for Neurodegenerative Disease. Nat. Rev. Neurol..

[5] Muddapu V.R., Dharshini S.A.P., Chakravarthy V.S., Gromiha M.M. (2020). Neurodegenerative Diseases—Is Metabolic Deficiency the Root Cause?. Front. Neurosci..

[6] Franklin H., Clarke B.E., Patani R. (2021). Astrocytes and Microglia in Neurodegenerative Diseases: Lessons from Human in Vitro Models. Prog. Neurobiol..

[7] Wilson D.M., Cookson M.R., Van Den Bosch L., Zetterberg H., Holtzman D.M., Dewachter I. (2023). Hallmarks of Neurodegenerative Diseases. Cell.

[8] Wei D.C., Morrison E.H. (2024). Histology, Astrocytes.

[9] Jha M.K., Kim J.-H., Song G.J., Lee W.-H., Lee I.-K., Lee H.-W., An S.S.A., Kim S., Suk K. (2018). Functional Dissection of Astrocyte-Secreted Proteins: Implications in Brain Health and Diseases. Prog. Neurobiol..

[10] Lee H.-G., Wheeler M.A., Quintana F.J. (2022). Function and Therapeutic Value of Astrocytes in Neurological Diseases. Nat. Rev. Drug Discov..

[11] Makarov M., Papa M., Korkotian E. (2024). Computational Modeling of Extrasynaptic NMDA Receptors: Insights into Dendritic Signal Amplification Mechanisms. Int. J. Mol. Sci..

[12] Kwon H.S., Koh S.-H. (2020). Neuroinflammation in Neurodegenerative Disorders: The Roles of Microglia and Astrocytes. Transl. Neurodegener..

[13] Verkhratsky A., Matteoli M., Parpura V., Mothet J., Zorec R. (2016). Astrocytes as Secretory Cells of the Central Nervous System: Idiosyncrasies of Vesicular Secretion. EMBO J..

[14] Matejuk A., Ransohoff R.M. (2020). Crosstalk Between Astrocytes and Microglia: An Overview. Front. Immunol..

[15] Vandenbark A.A., Offner H., Matejuk S., Matejuk A. (2021). Microglia and Astrocyte Involvement in Neurodegeneration and Brain Cancer. J. Neuroinflamm..

[16] Ahmad S., Srivastava R.K., Singh P., Naik U.P., Srivastava A.K. (2022). Role of Extracellular Vesicles in Glia-Neuron Intercellular Communication. Front. Mol. Neurosci..

[17] Verkhratsky A., Parpura V., Li B., Scuderi C., Li B., Parpura V., Verkhratsky A., Scuderi C. (2021). Astrocytes: The Housekeepers and Guardians of the CNS. Astrocytes in Psychiatric Disorders.

[18] Clavreul S., Dumas L., Loulier K. (2022). Astrocyte Development in the Cerebral Cortex: Complexity of Their Origin, Genesis, and Maturation. Front. Neurosci..

[19] Tumanova U.N., Savva O.V., Schegolev A.I. (2024). Brain Astrocytes: Classification, Morphological and Immunohistochemical Characteristics. CПHO (MPSE).

[20] Oberheim N.A., Goldman S.A., Nedergaard M., Milner R. (2012). Heterogeneity of Astrocytic Form and Function. Astrocytes.

[21] Cerrato V. (2020). Cerebellar Astrocytes: Much More Than Passive Bystanders in Ataxia Pathophysiology. J. Clin. Med..

[22] Kerstetter A.E., Miller R.H., Milner R. (2012). Isolation and Culture of Spinal Cord Astrocytes. Astrocytes.

[23] Li Q., Barres B.A. (2018). Microglia and Macrophages in Brain Homeostasis and Disease. Nat. Rev. Immunol..

[24] Althafar Z.M. (2022). Targeting Microglia in Alzheimer’s Disease: From Molecular Mechanisms to Potential Therapeutic Targets for Small Molecules. Molecules.

[25] Escartin C., Galea E., Lakatos A., O’Callaghan J.P., Petzold G.C., Serrano-Pozo A., Steinhäuser C., Volterra A., Carmignoto G., Agarwal A. (2021). Reactive Astrocyte Nomenclature, Definitions, and Future Directions. Nat. Neurosci..

[26] Edison P. (2024). Astroglial Activation: Current Concepts and Future Directions. Alzheimer’s Dement..

[27] Lawrence J.M., Schardien K., Wigdahl B., Nonnemacher M.R. (2023). Roles of Neuropathology-Associated Reactive Astrocytes: A Systematic Review. Acta Neuropathol. Commun..

[28] Ginhoux F., Lim S., Hoeffel G., Low D., Huber T. (2013). Origin and Differentiation of Microglia. Front. Cell. Neurosci..

[29] Dermitzakis I., Manthou M.E., Meditskou S., Tremblay M.-È., Petratos S., Zoupi L., Boziki M., Kesidou E., Simeonidou C., Theotokis P. (2023). Origin and Emergence of Microglia in the CNS—An Interesting (Hi)Story of an Eccentric Cell. Curr. Issues Mol. Biol..

[30] Cai Z., Hussain M.D., Yan L.-J. (2014). Microglia, Neuroinflammation, and Beta-Amyloid Protein in Alzheimer’s Disease. Int. J. Neurosci..

[31] Jurga A.M., Paleczna M., Kuter K.Z. (2020). Overview of General and Discriminating Markers of Differential Microglia Phenotypes. Front. Cell. Neurosci..

[32] Vidal-Itriago A., Radford R.A.W., Aramideh J.A., Maurel C., Scherer N.M., Don E.K., Lee A., Chung R.S., Graeber M.B., Morsch M. (2022). Microglia Morphophysiological Diversity and Its Implications for the CNS. Front. Immunol..

[33] Reddaway J., Richardson P.E., Bevan R.J., Stoneman J., Palombo M. (2023). Microglial Morphometric Analysis: So Many Options, so Little Consistency. Front. Neuroinform..

[34] Green T.R.F., Rowe R.K. (2024). Quantifying Microglial Morphology: An Insight into Function. Clin. Exp. Immunol..

[35] Lull M.E., Block M.L. (2010). Microglial Activation and Chronic Neurodegeneration. Neurotherapeutics.

[36] Luo X.-G., Chen S.-D. (2012). The Changing Phenotype of Microglia from Homeostasis to Disease. Transl. Neurodegener..

[37] Gómez-Budia M., Konttinen H., Saveleva L., Korhonen P., Jalava P.I., Kanninen K.M., Malm T. (2020). Glial Smog: Interplay between Air Pollution and Astrocyte-Microglia Interactions. Neurochem. Int..

[38] Li Y., Xu H., Wang H., Yang K., Luan J., Wang S. (2023). TREM2: Potential Therapeutic Targeting of Microglia for Alzheimer’s Disease. Biomed. Pharmacother..

[39] Stephenson J., Nutma E., Van Der Valk P., Amor S. (2018). Inflammation in CNS Neurodegenerative Diseases. Immunology.

[40] Oksanen M., Lehtonen S., Jaronen M., Goldsteins G., Hämäläinen R.H., Koistinaho J. (2019). Astrocyte Alterations in Neurodegenerative Pathologies and Their Modeling in Human Induced Pluripotent Stem Cell Platforms. Cell. Mol. Life Sci..

[41] Rock R.B., Gekker G., Hu S., Sheng W.S., Cheeran M., Lokensgard J.R., Peterson P.K. (2004). Role of Microglia in Central Nervous System Infections. Clin. Microbiol. Rev..

[42] Paolicelli R.C., Bergamini G., Rajendran L. (2019). Cell-to-Cell Communication by Extracellular Vesicles: Focus on Microglia. Neuroscience.

[43] Takenouchi T., Suzuki S., Shinkai H., Tsukimoto M., Sato M., Uenishi H., Kitani H. (2014). Extracellular ATP Does Not Induce P2X7 Receptor-Dependent Responses in Cultured Renal- and Liver-Derived Swine Macrophages. Results Immunol..

[44] Yang Y., Kim S.C., Yu T., Yi Y.-S., Rhee M.H., Sung G.-H., Yoo B.C., Cho J.Y. (2014). Functional Roles of P38 Mitogen-Activated Protein Kinase in Macrophage-Mediated Inflammatory Responses. Mediat. Inflamm..

[45] Rodríguez J.J., Witton J., Olabarria M., Noristani H.N., Verkhratsky A. (2010). Increase in the Density of Resting Microglia Precedes Neuritic Plaque Formation and Microglial Activation in a Transgenic Model of Alzheimer’s Disease. Cell Death Dis..

[46] Rodríguez J.J., Noristani H.N., Hilditch T., Olabarria M., Yeh C.Y., Witton J., Verkhratsky A. (2013). Increased Densities of Resting and Activated Microglia in the Dentate Gyrus Follow Senile Plaque Formation in the CA1 Subfield of the Hippocampus in the Triple Transgenic Model of Alzheimer’s Disease. Neurosci. Lett..

[47] Perry V.H., Holmes C. (2014). Microglial Priming in Neurodegenerative Disease. Nat. Rev. Neurol..

[48] Niraula A., Sheridan J.F., Godbout J.P. (2017). Microglia Priming with Aging and Stress. Neuropsychopharmacology.

[49] Makarov M., Kushnireva L., Papa M., Korkotian E. (2023). Presenilins and Mitochondria—An Intriguing Link: Mini-Review. Front. Neurosci..

[50] Zhang L., Zhang J., You Z. (2018). Switching of the Microglial Activation Phenotype Is a Possible Treatment for Depression Disorder. Front. Cell. Neurosci..

[51] Wang J., He W., Zhang J. (2023). A Richer and More Diverse Future for Microglia Phenotypes. Heliyon.

[52] Sun Z., Zhang X., So K.-F., Jiang W., Chiu K. (2024). Targeting Microglia in Alzheimer’s Disease: Pathogenesis and Potential Therapeutic Strategies. Biomolecules.

[53] Vainchtein I.D., Molofsky A.V. (2020). Astrocytes and Microglia: In Sickness and in Health. Trends Neurosci..

[54] Garland E.F., Hartnell I.J., Boche D. (2022). Microglia and Astrocyte Function and Communication: What Do We Know in Humans?. Front. Neurosci..

[55] Thakur S., Dhapola R., Sarma P., Medhi B., Reddy D.H. (2023). Neuroinflammation in Alzheimer’s Disease: Current Progress in Molecular Signaling and Therapeutics. Inflammation.

[56] Cui W., Sun C., Ma Y., Wang S., Wang X., Zhang Y. (2020). Inhibition of TLR4 Induces M2 Microglial Polarization and Provides Neuroprotection via the NLRP3 Inflammasome in Alzheimer’s Disease. Front. Neurosci..

[57] Fu Y., Kim H., Lee D.S., Han A., Heine H., Zamyatina A., Kim H.M. (2025). Structural Insight into TLR4/MD-2 Activation by Synthetic LPS Mimetics with Distinct Binding Modes. Nat. Commun..

[58] Lang G.-P., Li C., Han Y.-Y. (2021). Rutin Pretreatment Promotes Microglial M1 to M2 Phenotype Polarization. Neural Regen. Res..

[59] Milani P., Gagliardi S., Cova E., Cereda C. (2011). SOD1 Transcriptional and Posttranscriptional Regulation and Its Potential Implications in ALS. Neurol. Res. Int..

[60] Swanson K.V., Deng M., Ting J.P.-Y. (2019). The NLRP3 Inflammasome: Molecular Activation and Regulation to Therapeutics. Nat. Rev. Immunol..

[61] Agard N.J., Maltby D., Wells J.A. (2010). Inflammatory Stimuli Regulate Caspase Substrate Profiles. Mol. Cell. Proteom..

[62] Shi J., Zhao Y., Wang K., Shi X., Wang Y., Huang H., Zhuang Y., Cai T., Wang F., Shao F. (2015). Cleavage of GSDMD by Inflammatory Caspases Determines Pyroptotic Cell Death. Nature.

[63] Broz P., Dixit V.M. (2016). Inflammasomes: Mechanism of Assembly, Regulation and Signalling. Nat. Rev. Immunol..

[64] Di Virgilio F., Dal Ben D., Sarti A.C., Giuliani A.L., Falzoni S. (2017). The P2X7 Receptor in Infection and Inflammation. Immunity.

[65] Martínez-Frailes C., Di Lauro C., Bianchi C., De Diego-García L., Sebastián-Serrano Á., Boscá L., Díaz-Hernández M. (2019). Amyloid Peptide Induced Neuroinflammation Increases the P2X7 Receptor Expression in Microglial Cells, Impacting on Its Functionality. Front. Cell. Neurosci..

[66] Sarkar S., Rokad D., Malovic E., Luo J., Harischandra D.S., Jin H., Anantharam V., Huang X., Lewis M., Kanthasamy A. (2019). Manganese Activates NLRP3 Inflammasome Signaling and Propagates Exosomal Release of ASC in Microglial Cells. Sci. Signal..

[67] Heneka M.T., Kummer M.P., Stutz A., Delekate A., Schwartz S., Vieira-Saecker A., Griep A., Axt D., Remus A., Tzeng T.-C. (2013). NLRP3 Is Activated in Alzheimer’s Disease and Contributes to Pathology in APP/PS1 Mice. Nature.

[68] Oken A.C., Lisi N.E., Krishnamurthy I., McCarthy A.E., Godsey M.H., Glasfeld A., Mansoor S.E. (2024). High-Affinity Agonism at the P2X7 Receptor Is Mediated by Three Residues Outside the Orthosteric Pocket. Nat. Commun..

[69] Feofilaktova T., Kushnireva L., Segal M., Korkotian E. (2025). Calcium Signaling in Postsynaptic Mitochondria: Mechanisms, Dynamics, and Role in ATP Production. Front. Mol. Neurosci..

[70] Li T., Tan X., Li S., Al-Nusaif M., Le W. (2021). Role of Glia-Derived Extracellular Vesicles in Neurodegenerative Diseases. Front. Aging Neurosci..

[71] Liu W., Tang Y., Feng J. (2011). Cross Talk between Activation of Microglia and Astrocytes in Pathological Conditions in the Central Nervous System. Life Sci..

[72] Bernaus A., Blanco S., Sevilla A. (2020). Glia Crosstalk in Neuroinflammatory Diseases. Front. Cell. Neurosci..

[73] Chavda V., Singh K., Patel V., Mishra M., Mishra A.K. (2022). Neuronal Glial Crosstalk: Specific and Shared Mechanisms in Alzheimer’s Disease. Brain Sci..

[74] Patani R., Hardingham G.E., Liddelow S.A. (2023). Functional Roles of Reactive Astrocytes in Neuroinflammation and Neurodegeneration. Nat. Rev. Neurol..

[75] Silvin A., Qian J., Ginhoux F. (2023). Brain Macrophage Development, Diversity and Dysregulation in Health and Disease. Cell. Mol. Immunol..

[76] Argaw A.T., Asp L., Zhang J., Navrazhina K., Pham T., Mariani J.N., Mahase S., Dutta D.J., Seto J., Kramer E.G. (2012). Astrocyte-Derived VEGF-A Drives Blood-Brain Barrier Disruption in CNS Inflammatory Disease. J. Clin. Investig..

[77] Peferoen L., Kipp M., Van Der Valk P., Van Noort J.M., Amor S. (2014). Oligodendrocyte-microglia Cross-talk in the Central Nervous System. Immunology.

[78] Domingues H.S., Portugal C.C., Socodato R., Relvas J.B. (2016). Oligodendrocyte, Astrocyte, and Microglia Crosstalk in Myelin Development, Damage, and Repair. Front. Cell Dev. Biol..

[79] Kalafatakis I., Karagogeos D. (2021). Oligodendrocytes and Microglia: Key Players in Myelin Development, Damage and Repair. Biomolecules.

[80] Fahrenhold M., Rakic S., Classey J., Brayne C., Ince P.G., Nicoll J.A.R., Boche D. (2018). MRC-CFAS TREM2 Expression in the Human Brain: A Marker of Monocyte Recruitment?. Brain Pathol..

[81] Ruganzu J.B., Zheng Q., Wu X., He Y., Peng X., Jin H., Zhou J., Ma R., Ji S., Ma Y. (2021). TREM2 Overexpression Rescues Cognitive Deficits in APP/PS1 Transgenic Mice by Reducing Neuroinflammation via the JAK/STAT/SOCS Signaling Pathway. Exp. Neurol..

[82] Van Lengerich B., Zhan L., Xia D., Chan D., Joy D., Park J.I., Tatarakis D., Calvert M., Hummel S., Lianoglou S. (2023). A TREM2-Activating Antibody with a Blood–Brain Barrier Transport Vehicle Enhances Microglial Metabolism in Alzheimer’s Disease Models. Nat. Neurosci..

[83] Miao J., Ma H., Yang Y., Liao Y., Lin C., Zheng J., Yu M., Lan J. (2023). Microglia in Alzheimer’s Disease: Pathogenesis, Mechanisms, and Therapeutic Potentials. Front. Aging Neurosci..

[84] Hu X., Li J., Fu M., Zhao X., Wang W. (2021). The JAK/STAT Signaling Pathway: From Bench to Clinic. Signal Transduct. Target. Ther..

[85] Heinrich P.C., Behrmann I., Haan S., Hermanns H.M., Müller-Newen G., Schaper F. (2003). Principles of Interleukin (IL)-6-Type Cytokine Signalling and Its Regulation. Biochem. J..

[86] Yu H., Pardoll D., Jove R. (2009). STATs in Cancer Inflammation and Immunity: A Leading Role for STAT3. Nat. Rev. Cancer.

[87] Giunti D., Parodi B., Cordano C., Uccelli A., Kerlero De Rosbo N. (2014). Can We Switch Microglia’s Phenotype to Foster Neuroprotection? Focus on Multiple Sclerosis. Immunology.

[88] Tang Y., Le W. (2016). Differential Roles of M1 and M2 Microglia in Neurodegenerative Diseases. Mol. Neurobiol..

[89] Diniz L.P., Tortelli V., Matias I., Morgado J., Araujo A.P.B., Melo H.M., da Silva G.S.S., Alves-Leon S.V., De Souza J.M., Ferreira S.T. (2017). Astrocyte Transforming Growth Factor Beta 1 Protects Synapses against Aβ Oligomers in Alzheimer’s Disease Model. J. Neurosci..

[90] Cai Y., Liu J., Wang B., Sun M., Yang H. (2022). Microglia in the Neuroinflammatory Pathogenesis of Alzheimer’s Disease and Related Therapeutic Targets. Front. Immunol..

[91] Jian M., Kwan J.S.-C., Bunting M., Ng R.C.-L., Chan K.H. (2019). Adiponectin Suppresses Amyloid-β Oligomer (AβO)-Induced Inflammatory Response of Microglia via AdipoR1-AMPK-NF-κB Signaling Pathway. J. Neuroinflamm..

[92] Chuang K.-A., Li M.-H., Lin N.-H., Chang C.-H., Lu I.-H., Pan I.-H., Takahashi T., Perng M.-D., Wen S.-F. (2017). Rhinacanthin C Alleviates Amyloid-*β* Fibrils’ Toxicity on Neurons and Attenuates Neuroinflammation Triggered by LPS, Amyloid-*β*, and Interferon-*γ* in Glial Cells. Oxidative Med. Cell. Longev..

[93] Gomes B.A.Q., Silva J.P.B., Romeiro C.F.R., Dos Santos S.M., Rodrigues C.A., Gonçalves P.R., Sakai J.T., Mendes P.F.S., Varela E.L.P., Monteiro M.C. (2018). Neuroprotective Mechanisms of Resveratrol in Alzheimer’s Disease: Role of SIRT1. Oxidative Med. Cell. Longev..

[94] Singh V., Ubaid S. (2020). Role of Silent Information Regulator 1 (SIRT1) in Regulating Oxidative Stress and Inflammation. Inflammation.

[95] Stratoulias V., Venero J.L., Tremblay M., Joseph B. (2019). Microglial Subtypes: Diversity within the Microglial Community. EMBO J..

[96] Candlish M., Hefendehl J.K. (2021). Microglia Phenotypes Converge in Aging and Neurodegenerative Disease. Front. Neurol..

[97] Wei Y., Li X. (2022). Different Phenotypes of Microglia in Animal Models of Alzheimer Disease. Immun. Ageing.

[98] Sondag C.M., Dhawan G., Combs C.K. (2009). Beta Amyloid Oligomers and Fibrils Stimulate Differential Activation of Primary Microglia. J. Neuroinflamm..

[99] Srivastava S., Ahmad R., Khare S.K. (2021). Alzheimer’s Disease and Its Treatment by Different Approaches: A Review. Eur. J. Med. Chem..

[100] Pascoal T.A., Benedet A.L., Ashton N.J., Kang M.S., Therriault J., Chamoun M., Savard M., Lussier F.Z., Tissot C., Karikari T.K. (2021). Microglial Activation and Tau Propagate Jointly across Braak Stages. Nat. Med..

[101] Garcia-Contreras M., Thakor A. (2023). Extracellular Vesicles in Alzheimer’s Disease: From Pathology to Therapeutic Approaches. Neural Regen. Res..

[102] Joshi P., Turola E., Ruiz A., Bergami A., Libera D.D., Benussi L., Giussani P., Magnani G., Comi G., Legname G. (2014). Microglia Convert Aggregated Amyloid-β into Neurotoxic Forms through the Shedding of Microvesicles. Cell Death Differ..

[103] Elsherbini A., Kirov A.S., Dinkins M.B., Wang G., Qin H., Zhu Z., Tripathi P., Crivelli S.M., Bieberich E. (2020). Association of Aβ with Ceramide-Enriched Astrosomes Mediates Aβ Neurotoxicity. Acta Neuropathol. Commun..

[104] Elsherbini A., Qin H., Zhu Z., Tripathi P., Crivelli S.M., Bieberich E. (2020). In Vivo Evidence of Exosome-Mediated Aβ Neurotoxicity. Acta Neuropathol. Commun..

[105] Clayton K., Delpech J.C., Herron S., Iwahara N., Ericsson M., Saito T., Saido T.C., Ikezu S., Ikezu T. (2021). Plaque Associated Microglia Hyper-Secrete Extracellular Vesicles and Accelerate Tau Propagation in a Humanized APP Mouse Model. Mol. Neurodegener..

[106] Muraoka S., Lin W., Takamatsu-Yukawa K., Hu J., Ikezu S., DeTure M.A., Dickson D.W., Emili A., Ikezu T. (2021). Enrichment of Phosphorylated Tau (Thr181) and Functionally Interacting Molecules in Chronic Traumatic Encephalopathy Brain-Derived Extracellular Vesicles. Aging Dis..

[107] Butterfield D.A., Boyd-Kimball D. (2004). Amyloid β-Peptide(1–42) Contributes to the Oxidative Stress and Neurodegeneration Found in Alzheimer Disease Brain. Brain Pathol..

[108] Kozin S.A. (2023). Role of Interaction between Zinc and Amyloid Beta in Pathogenesis of Alzheimer’s Disease. Biochemistry.

[109] Lauro C., Catalano M., Trettel F., Mainiero F., Ciotti M.T., Eusebi F., Limatola C. (2006). The Chemokine CX3CL1 Reduces Migration and Increases Adhesion of Neurons with Mechanisms Dependent on the Β1 Integrin Subunit. J. Immunol..

[110] Raman D., Sobolik-Delmaire T., Richmond A. (2011). Chemokines in Health and Disease. Exp. Cell Res..

[111] Aguzzi A., Barres B.A., Bennett M.L. (2013). Microglia: Scapegoat, Saboteur, or Something Else?. Science.

[112] Pawelec P., Ziemka-Nalecz M., Sypecka J., Zalewska T. (2020). The Impact of the CX3CL1/CX3CR1 Axis in Neurological Disorders. Cells.

[113] Puntambekar S.S., Moutinho M., Lin P.B.-C., Jadhav V., Tumbleson-Brink D., Balaji A., Benito M.A., Xu G., Oblak A., Lasagna-Reeves C.A. (2022). CX3CR1 Deficiency Aggravates Amyloid Driven Neuronal Pathology and Cognitive Decline in Alzheimer’s Disease. Mol. Neurodegener..

[114] Cho S.-H., Sun B., Zhou Y., Kauppinen T.M., Halabisky B., Wes P., Ransohoff R.M., Gan L. (2011). CX3CR1 Protein Signaling Modulates Microglial Activation and Protects against Plaque-Independent Cognitive Deficits in a Mouse Model of Alzheimer Disease. J. Biol. Chem..

[115] Winter A.N., Subbarayan M.S., Grimmig B., Weesner J.A., Moss L., Peters M., Weeber E., Nash K., Bickford P.C. (2020). Two Forms of CX3CL1 Display Differential Activity and Rescue Cognitive Deficits in CX3CL1 Knockout Mice. J. Neuroinflamm..

[116] Bolós M., Llorens-Martín M., Perea J.R., Jurado-Arjona J., Rábano A., Hernández F., Avila J. (2017). Absence of CX3CR1 Impairs the Internalization of Tau by Microglia. Mol. Neurodegener..

[117] Ulrich J.D., Ulland T.K., Colonna M., Holtzman D.M. (2017). Elucidating the Role of TREM2 in Alzheimer’s Disease. Neuron.

[118] Wang Y., Wang L., Zhan H., Luo X., Zeng Y., Wu W., Zhang X., Wang F. (2020). TREM2 Ameliorates Neuroinflammatory Response and Cognitive Impairment via PI3K/AKT/FoxO3a Signaling Pathway in Alzheimer’s disease mice. Aging.

[119] Wang Q., Yang W., Zhang J., Zhao Y., Xu Y. (2020). TREM2 Overexpression Attenuates Cognitive Deficits in Experimental Models of Vascular Dementia. Neural Plast..

[120] Price B.R., Sudduth T.L., Weekman E.M., Johnson S., Hawthorne D., Woolums A., Wilcock D.M. (2020). Therapeutic Trem2 Activation Ameliorates Amyloid-Beta Deposition and Improves Cognition in the 5XFAD Model of Amyloid Deposition. J. Neuroinflamm..

[121] Lee C.Y.D., Daggett A., Gu X., Jiang L.-L., Langfelder P., Li X., Wang N., Zhao Y., Park C.S., Cooper Y. (2018). Elevated TREM2 Gene Dosage Reprograms Microglia Responsivity and Ameliorates Pathological Phenotypes in Alzheimer’s Disease Models. Neuron.

[122] Parhizkar S., Arzberger T., Brendel M., Kleinberger G., Deussing M., Focke C., Nuscher B., Xiong M., Ghasemigharagoz A., Katzmarski N. (2019). Loss of TREM2 Function Increases Amyloid Seeding but Reduces Plaque-Associated ApoE. Nat. Neurosci..

[123] Schlepckow K., Monroe K.M., Kleinberger G., Cantuti-Castelvetri L., Parhizkar S., Xia D., Willem M., Werner G., Pettkus N., Brunner B. (2020). Enhancing Protective Microglial Activities with a Dual Function TREM 2 Antibody to the Stalk Region. EMBO Mol. Med..

[124] Wang S., Sudan R., Peng V., Zhou Y., Du S., Yuede C.M., Lei T., Hou J., Cai Z., Cella M. (2022). TREM2 Drives Microglia Response to Amyloid-β via SYK-Dependent and -Independent Pathways. Cell.

[125] Mócsai A., Ruland J., Tybulewicz V.L.J. (2010). The SYK Tyrosine Kinase: A Crucial Player in Diverse Biological Functions. Nat. Rev. Immunol..

[126] Billadeau D.D., Upshaw J.L., Schoon R.A., Dick C.J., Leibson P.J. (2003). NKG2D-DAP10 Triggers Human NK Cell–Mediated Killing via a Syk-Independent Regulatory Pathway. Nat. Immunol..

[127] Lanier L.L. (2009). DAP10- and DAP12-associated Receptors in Innate Immunity. Immunol. Rev..

[128] Krasemann S., Madore C., Cialic R., Baufeld C., Calcagno N., El Fatimy R., Beckers L., O’Loughlin E., Xu Y., Fanek Z. (2017). The TREM2-APOE Pathway Drives the Transcriptional Phenotype of Dysfunctional Microglia in Neurodegenerative Diseases. Immunity.

[129] Bradshaw E.M., Chibnik L.B., Keenan B.T., Ottoboni L., Raj T., Tang A., Rosenkrantz L.L., Imboywa S., Lee M., Von Korff A. (2013). CD33 Alzheimer’s Disease Locus: Altered Monocyte Function and Amyloid Biology. Nat. Neurosci..

[130] Miles L.A., Hermans S.J., Crespi G.A.N., Gooi J.H., Doughty L., Nero T.L., Markulić J., Ebneth A., Wroblowski B., Oehlrich D. (2019). Small Molecule Binding to Alzheimer Risk Factor CD33 Promotes Aβ Phagocytosis. iScience.

[131] Griciuc A., Federico A.N., Natasan J., Forte A.M., McGinty D., Nguyen H., Volak A., LeRoy S., Gandhi S., Lerner E.P. (2020). Gene Therapy for Alzheimer’s Disease Targeting CD33 Reduces Amyloid Beta Accumulation and Neuroinflammation. Hum. Mol. Genet..

[132] Griciuc A., Patel S., Federico A.N., Choi S.H., Innes B.J., Oram M.K., Cereghetti G., McGinty D., Anselmo A., Sadreyev R.I. (2019). TREM2 Acts Downstream of CD33 in Modulating Microglial Pathology in Alzheimer’s Disease. Neuron.

[133] Mendsaikhan A., Tooyama I., Walker D.G. (2019). Microglial Progranulin: Involvement in Alzheimer’s Disease and Neurodegenerative Diseases. Cells.

[134] Rhinn H., Tatton N., McCaughey S., Kurnellas M., Rosenthal A. (2022). Progranulin as a Therapeutic Target in Neurodegenerative Diseases. Trends Pharmacol. Sci..

[135] Minami S.S., Min S.-W., Krabbe G., Wang C., Zhou Y., Asgarov R., Li Y., Martens L.H., Elia L.P., Ward M.E. (2014). Progranulin Protects against Amyloid β Deposition and Toxicity in Alzheimer’s Disease Mouse Models. Nat. Med..

[136] Yu H., Xiong M., Zhang Z. (2023). The Role of Glycogen Synthase Kinase 3 Beta in Neurodegenerative Diseases. Front. Mol. Neurosci..

[137] Gupta N., Shyamasundar S., Patnala R., Karthikeyan A., Arumugam T.V., Ling E.-A., Dheen S.T. (2018). Recent Progress in Therapeutic Strategies for Microglia-Mediated Neuroinflammation in Neuropathologies. Expert Opin. Ther. Targets.

[138] Sofroniew M.V. (2015). Astrogliosis. Cold Spring Harb. Perspect. Biol..

[139] Wang H., Song G., Chuang H., Chiu C., Abdelmaksoud A., Ye Y., Zhao L. (2018). Portrait of Glial Scar in Neurological Diseases. Int. J. Immunopathol. Pharmacol..

[140] D’Ambrosi N., Apolloni S. (2020). Fibrotic Scar in Neurodegenerative Diseases. Front. Immunol..

[141] Paidlewar M., Kumari S., Dhapola R., Sharma P., HariKrishnaReddy D. (2024). Unveiling the Role of Astrogliosis in Alzheimer’s Disease Pathology: Insights into Mechanisms and Therapeutic Approaches. Int. Immunopharmacol..

[142] Saadoun S., Papadopoulos M.C., Watanabe H., Yan D., Manley G.T., Verkman A.S. (2005). Involvement of Aquaporin-4 in Astroglial Cell Migration and Glial Scar Formation. J. Cell Sci..

[143] Koyama Y. (2014). Signaling Molecules Regulating Phenotypic Conversions of Astrocytes and Glial Scar Formation in Damaged Nerve Tissues. Neurochem. Int..

[144] Bao Y., Qin L., Kim E., Bhosle S., Guo H., Febbraio M., Haskew-Layton R.E., Ratan R., Cho S. (2012). CD36 Is Involved in Astrocyte Activation and Astroglial Scar Formation. J. Cereb. Blood Flow Metab..

[145] Anderson M.A., Burda J.E., Ren Y., Ao Y., O’Shea T.M., Kawaguchi R., Coppola G., Khakh B.S., Deming T.J., Sofroniew M.V. (2016). Astrocyte Scar Formation Aids Central Nervous System Axon Regeneration. Nature.

[146] Streeter K.A., Sunshine M.D., Brant J.O., Sandoval A.G.W., Maden M., Fuller D.D. (2020). Molecular and Histologic Outcomes Following Spinal Cord Injury in Spiny Mice, *Acomys cahirinus*. J. Comp. Neurol..

[147] Nogueira-Rodrigues J., Leite S.C., Pinto-Costa R., Sousa S.C., Luz L.L., Sintra M.A., Oliveira R., Monteiro A.C., Pinheiro G.G., Vitorino M. (2022). Rewired Glycosylation Activity Promotes Scarless Regeneration and Functional Recovery in Spiny Mice after Complete Spinal Cord Transection. Dev. Cell.

[148] Yin J.-C., Zhang L., Ma N.-X., Wang Y., Lee G., Hou X.-Y., Lei Z.-F., Zhang F.-Y., Dong F.-P., Wu G.-Y. (2019). Chemical Conversion of Human Fetal Astrocytes into Neurons through Modulation of Multiple Signaling Pathways. Stem Cell Rep..

[149] Ma Y., Xie H., Du X., Wang L., Jin X., Zhang Q., Han Y., Sun S., Wang L., Li X. (2021). In Vivo Chemical Reprogramming of Astrocytes into Neurons. Cell Discov..

[150] Guo Z., Zhang L., Wu Z., Chen Y., Wang F., Chen G. (2014). In Vivo Direct Reprogramming of Reactive Glial Cells into Functional Neurons after Brain Injury and in an Alzheimer’s Disease Model. Cell Stem Cell.

[151] Liu Y., Guo J., Matoga M., Korotkova M., Jakobsson P.-J., Aguzzi A. (2024). NG2 Glia Protect against Prion Neurotoxicity by Inhibiting Microglia-to-Neuron Prostaglandin E2 Signaling. Nat. Neurosci..

[152] Nielsen H.M., Ek D., Avdic U., Orbjörn C., Hansson O., Veerhuis R., Rozemuller A.J., Brun A., Minthon L., The Netherlands Brain Bank (2013). NG2 Cells, a New Trail for Alzheimer’s Disease Mechanisms?. Acta Neuropathol. Commun..

[153] Vasic V., Barth K., Schmidt M.H.H. (2019). Neurodegeneration and Neuro-Regeneration—Alzheimer’s Disease and Stem Cell Therapy. IJMS.

[154] Yavarpour-Bali H., Ghasemi-Kasman M., Shojaei A. (2020). Direct Reprogramming of Terminally Differentiated Cells into Neurons: A Novel and Promising Strategy for Alzheimer’s Disease Treatment. Prog. Neuro-Psychopharmacol. Biol. Psychiatry.

[155] Wang F., Cheng L., Zhang X. (2021). Reprogramming Glial Cells into Functional Neurons for Neuro-Regeneration: Challenges and Promise. Neurosci. Bull..

[156] Liu Y., Wei C., Yang Y., Zhu Z., Ren Y., Pi R. (2024). In Situ Chemical Reprogramming of Astrocytes into Neurons: A New Hope for the Treatment of Central Neurodegenerative Diseases?. Eur. J. Pharmacol..

[157] Li Y., Xia X., Wang Y., Zheng J.C. (2022). Mitochondrial Dysfunction in Microglia: A Novel Perspective for Pathogenesis of Alzheimer’s Disease. J. Neuroinflamm..

[158] Agrawal I., Jha S. (2020). Mitochondrial Dysfunction and Alzheimer’s Disease: Role of Microglia. Front. Aging Neurosci..

[159] Park J., Choi H., Min J., Park S., Kim J., Park H., Kim B., Chae J., Yim M., Lee D. (2013). Mitochondrial Dynamics Modulate the Expression of Pro-inflammatory Mediators in Microglial Cells. J. Neurochem..

[160] Ye J., Jiang Z., Chen X., Liu M., Li J., Liu N. (2017). The Role of Autophagy in Pro-inflammatory Responses of Microglia Activation via Mitochondrial Reactive Oxygen Species In Vitro. J. Neurochem..

[161] Chiurazzi M., Di Maro M., Cozzolino M., Colantuoni A. (2020). Mitochondrial Dynamics and Microglia as New Targets in Metabolism Regulation. Int. J. Mol. Sci..

[162] Joshi A.U., Minhas P.S., Liddelow S.A., Haileselassie B., Andreasson K.I., Dorn G.W., Mochly-Rosen D. (2019). Fragmented Mitochondria Released from Microglia Trigger A1 Astrocytic Response and Propagate Inflammatory Neurodegeneration. Nat. Neurosci..

[163] Mulica P., Grünewald A., Pereira S.L. (2021). Astrocyte-Neuron Metabolic Crosstalk in Neurodegeneration: A Mitochondrial Perspective. Front. Endocrinol..

[164] Gollihue J.L., Norris C.M. (2020). Astrocyte Mitochondria: Central Players and Potential Therapeutic Targets for Neurodegenerative Diseases and Injury. Ageing Res. Rev..

[165] Araujo A.P.B., Vargas G., Hayashide L.D.S., Matias I., Andrade C.B.V., De Carvalho J.J., Gomes F.C.A., Diniz L.P. (2024). Aging Promotes an Increase in Mitochondrial Fragmentation in Astrocytes. Front. Cell. Neurosci..

[166] Mi Y., Qi G., Vitali F., Shang Y., Raikes A.C., Wang T., Jin Y., Brinton R.D., Gu H., Yin F. (2023). Loss of Fatty Acid Degradation by Astrocytic Mitochondria Triggers Neuroinflammation and Neurodegeneration. Nat. Metab..

[167] Dematteis G., Vydmantaitė G., Ruffinatti F.A., Chahin M., Farruggio S., Barberis E., Ferrari E., Marengo E., Distasi C., Morkūnienė R. (2020). Proteomic Analysis Links Alterations of Bioenergetics, Mitochondria-ER Interactions and Proteostasis in Hippocampal Astrocytes from 3xTg-AD Mice. Cell Death Dis..

[168] Gong C., Bonfili L., Zheng Y., Cecarini V., Cuccioloni M., Angeletti M., Dematteis G., Tapella L., Genazzani A.A., Lim D. (2023). Immortalized Alzheimer’s Disease Astrocytes: Characterization of Their Proteolytic Systems. Mol. Neurobiol..

[169] Dematteis G., Tapella L., Lim D. (2024). Probing the Endoplasmic Reticulum-Mitochondria Interaction in Alzheimer’s Disease: Searching Far and Wide. Neural Regen. Res..

[170] Górska A., Markiewicz-Gospodarek A., Markiewicz R., Chilimoniuk Z., Borowski B., Trubalski M., Czarnek K. (2023). Distribution of Iron, Copper, Zinc and Cadmium in Glia, Their Influence on Glial Cells and Relationship with Neurodegenerative Diseases. Brain Sci..

[171] Chen L., Shen Q., Liu Y., Zhang Y., Sun L., Ma X., Song N., Xie J. (2025). Homeostasis and Metabolism of Iron and Other Metal Ions in Neurodegenerative Diseases. Signal Transduct. Target. Ther..

[172] Gromadzka G., Wilkaniec A., Tarnacka B., Hadrian K., Bendykowska M., Przybyłkowski A., Litwin T. (2024). The Role of Glia in Wilson’s Disease: Clinical, Neuroimaging, Neuropathological and Molecular Perspectives. Int. J. Mol. Sci..

[173] Wang M., Tang G., Zhou C., Guo H., Hu Z., Hu Q., Li G. (2023). Revisiting the Intersection of Microglial Activation and Neuroinflammation in Alzheimer’s Disease from the Perspective of Ferroptosis. Chem.-Biol. Interact..

[174] Blasco-Agell L., Pons-Espinal M., Testa V., Roch G., Montero-Muñoz J., Fernandez-Carasa I., Baruffi V., Gonzalez-Sepulveda M., Richaud-Patin Y., Jimenez S. (2025). LRRK2-Mutant Microglia and Neuromelanin Synergize to Drive Dopaminergic Neurodegeneration in an iPSC-Based Parkinson’s Disease Model. Commun. Biol..

[175] Moreno-García A., Kun A., Calero M., Calero O. (2021). The Neuromelanin Paradox and Its Dual Role in Oxidative Stress and Neurodegeneration. Antioxidants.

[176] Volpicelli-Daley L. (2023). Neuromelanin as a Nidus for Neurodegeneration. Brain.

[177] Zucca F.A., Basso E., Cupaioli F.A., Ferrari E., Sulzer D., Casella L., Zecca L. (2014). Neuromelanin of the Human Substantia Nigra: An Update. Neurotox. Res..

[178] Kim S., Chun H., Kim Y., Kim Y., Park U., Chu J., Bhalla M., Choi S.-H., Yousefian-Jazi A., Kim S. (2024). Astrocytic Autophagy Plasticity Modulates Aβ Clearance and Cognitive Function in Alzheimer’s Disease. Mol. Neurodegener..

[179] Samokhina E., Mangat A., Malladi C.S., Gyengesi E., Morley J.W., Buskila Y. (2025). Potassium Homeostasis during Disease Progression of Alzheimer’s Disease. J. Physiol..

[180] Hancock S.M., Finkelstein D.I., Adlard P.A. (2014). Glia and Zinc in Ageing and Alzheimer’s Disease: A Mechanism for Cognitive Decline?. Front. Aging Neurosci..

[181] Escalada P., Ezkurdia A., Ramírez M.J., Solas M. (2024). Essential Role of Astrocytes in Learning and Memory. Int. J. Mol. Sci..

[182] Williamson M.R., Kwon W., Woo J., Ko Y., Maleki E., Yu K., Murali S., Sardar D., Deneen B. (2024). Learning-Associated Astrocyte Ensembles Regulate Memory Recall. Nature.

[183] Wang C., Wang L., Gu Y. (2021). Microglia, Synaptic Dynamics and Forgetting. Brain Res. Bull..

[184] Enomoto S., Kato T.A. (2021). Involvement of Microglia in Disturbed Fear Memory Regulation: Possible Microglial Contribution to the Pathophysiology of Posttraumatic Stress Disorder. Neurochem. Int..

